# Identifying gaps on health impacts, exposures, and vulnerabilities to climate change on human health and wellbeing in South America: a scoping review

**DOI:** 10.1016/j.lana.2023.100580

**Published:** 2023-08-24

**Authors:** Yasna K. Palmeiro-Silva, Andres G. Lescano, Elaine C. Flores, Yamileth Astorga E, Luciana Rojas, Mario G. Chavez, Wendel Mora-Rivera, Stella M. Hartinger

**Affiliations:** a Institute for Global Health, University College London, London, United Kingdom; b Centro de Políticas Públicas, Pontificia Universidad Católica de Chile, Santiago, Chile; c Clima, Latin American Center of Excellence for Climate Change and Health, Universidad Peruana Cayetano Heredia, Lima, Peru; d Emerge, Emerging Diseases and Climate Change Research Unit, School of Public Health and Administration, Universidad Peruana Cayetano Heredia, Lima, Peru; e Centre on Climate Change and Planetary Health, London School of Hygiene and Tropical Medicine, London, United Kingdom; f The Stanford Center for Innovation in Global Health, Stanford University, Stanford, CA, USA; g Escuela de Tecnologías en Salud, Universidad de Costa Rica, San Pedro, San José, Costa Rica; h Universidad Peruana Cayetano Heredia, Lima, Peru; i Sociedad Científica de San Fernando, Universidad Nacional Mayor de San Marcos, Lima, Peru; j InterAmerican Center for Global Health (CISG), Puntarenas, Costa Rica; k Escuela de Enfermería, Universidad Latina de Costa Rica, San José, Costa Rica

**Keywords:** Climate change, South America, Health, Human impacts, Environmental exposure, Vulnerable populations, Global health

## Abstract

There is an important gap in regional information on climate change and health, limiting the development of science-based climate policies in South American countries. This study aims to identify the main gaps in the existing scientific literature on the impacts, exposure, and vulnerabilities of climate change on population health. A scoping review was performed guided by four sub-questions focused on the impacts of climate change on physical and mental health, exposure and vulnerability factors of population to climate hazards. The main findings showed that physical impacts mainly included infectious diseases, while mental health impacts included trauma, depression, and anxiety. Evidence on population exposure to climate hazards is limited, and social determinants of health and individual factors were identified as vulnerability factors. Overall, evidence on the intersection between climate change and health is limited in South America and has been generated in silos, with limited transdisciplinary research. More formal and systematic information should be generated to inform public policy.

**Funding:**

None.

## Introduction

Anthropogenic climate change, understood as “*a change in the state of the climate that can be identified by changes in the mean and/or the variability of its properties and that persists for an extended period, typically decades or longer*” and due to “*persistent anthropogenic changes in the composition of the atmosphere or in land use*”,^1^ is threatening the health and wellbeing of South American populations by increasing the risk of climate-sensitive temperature-, flood-, and drought-related morbidity and mortality; fresh water and food insecurity; and infectious diseases, especially those related to mosquito transmission.^1^^,^^2^ Although anthropogenic climate change is a global phenomenon, South American countries are highly vulnerable at different levels due to their limited preparedness and capacity to respond to these climate hazards, together with fragile and under-resourced healthcare systems, as well as structural social inequities.^2^, ^3^, ^4^, ^5^

Despite the growing global evidence on climate hazards and their impacts on human health and wellbeing, global estimates often hide significant differences at regional and local levels. For example, high temperatures and more extreme weather events might be present in different cities worldwide; however, the impact on human populations is mediated by social vulnerability factors or individual susceptibilities, including poorly planned urban and peri-urban features, high prevalence of pre-existing comorbidities, and marginalised groups including indigenous populations, which tend to increase the risk of negative health outcomes within populations.^6^^,^^7^ Therefore, to understand the magnitude of climate change impacts on population health and identify the most affected populations, more detailed and country-specific analyses are needed to adequately inform the public, policymakers, the media, and key stakeholders.^8^

Unfortunately, regional and sub-regional information on the relationship between climate change and population health has been mainly generated in high-income countries, including Australia, Canada, those in Europe, and the United States of America.^9^ This situation creates significant knowledge gaps in low and middle-income countries, including those from South America (SA). Additionally, existing evidence mainly focuses on specific countries, lacking a broader and systemic perspective of the situation in the region.^9^ This lack of evidence and insights might affect and limit the decision-making capacity and impair climate adaptation and mitigation policies. To address this issue, the Lancet Countdown South America (LCSA) aims to review and discuss the current regional evidence regarding health and climate change across five key domains (*i*) health hazards, exposures, and impacts; (*ii*) adaptation, planning, and resilience for health; (*iii*) mitigation actions and health co-benefits; (*iv*) economics and finance; and (*v*) public and political engagement.

This scoping review aims to identify the main gaps in the existing scientific literature on the impacts of anthropogenic climate change on human health and wellbeing in South American populations, the degree of human populations exposure to climate change hazards, and the main vulnerability and/or susceptibility factors to climate hazards that could increase the risk of climate change adverse impacts on health and wellbeing. This information will be useful to inform future research and policy aimed at promoting adaptation and resilience to climate change, as well as preventing and ameliorating the projected climate-related health impacts.

## Methods

### Design and search strategies

This scoping review (ScR) followed the Arksey and O’Malley framework.^10^ The overarching research question was *“What is the scientific evidence on the impacts, exposure, and vulnerabilities to climate change hazards on human health and wellbeing in South America?”* From this question, four specific sub-questions were proposed to simplify the searches and to focus the identification of more specific knowledge gaps:

- RsQ1: *What are the main impacts of climate change hazards on physical health and wellbeing in South America?*
- RsQ2: *What are the main impacts of climate change hazards on mental health and wellbeing in South America?*
- RsQ3: *To what extent human populations in South America are exposed to the hazards of climate change.*
- RsQ4: *What are the main vulnerabilities or susceptibility factors present in the South American human population groups that could increase the risk of climate change adverse impacts on health and wellbeing?*

Different search strategies were defined for each research question (Supplementary Tables S1–S4 in the Supplementary Material). The understanding of the concepts involved in each research sub-question is based on the definitions of the Lancet Countdown global reports.^11^, ^12^, ^13^, ^14^ Searches were conducted using keywords and synonyms in English.

Given that the purpose was to retrieve scientific evidence relevant to South America, the following databases were considered: Web of Science, PubMed, PubMed (MeSH), ProQuest, Scopus, SciELO, and BIREME/LILACS. All these databases complement each other to deliver information from multidisciplinary, biomedical, and regional databases.

The searches were performed between October and November 2021. The identified references in every database were downloaded and then uploaded to Rayyan® online manager. All duplicates were removed, and the final references were analysed.

This review did not need ethical approval as it worked with publicly available, secondary data.

### Selection of studies

The selection of studies was independently done by two reviewers (LR, MGC, or WM) following two main steps. First, titles and abstracts were analysed looking for articles that could provide relevant information to answer the research questions. Second, from the selected titles and abstracts, full texts were searched and analysed looking for articles that provided information on *i*) impacts of climate change on human health and wellbeing, *ii*) the degree of exposure to climate hazards, or *iii*) the main vulnerability factors that increase the risk of negative impacts from climate change. The selection of articles was restricted to South American countries only. Any disagreements between the reviewers were resolved by a third reviewer (YKPS, AGL, EF, or YA) who analysed the records and made a final decision.

Studies were included if they were published in Spanish, English, or Portuguese, and included information on the impacts, exposure, and vulnerabilities of climate change on population health in South America. There were no restrictions for publication dates. Studies were excluded if they were focused on non-human animals; opinion articles; or did not analyse the link between climate change impacts, exposures, or vulnerabilities and population health.

### Data extraction process

Two reviewers (LR, MGC, or WM) independently extracted all relevant information from full texts using a standardised form. This form collected the following information: title of the article; year of publication; authors; type of publication; a brief context of the study; the aim of the article; country and/or region of study; main characteristics of the methodology; and main findings. A group of third reviewers (YKPS, AGL, EF, and YA) evaluated the consistency of data extraction and checked any necessary re-revision of information from the extracted articles.

### Critical appraisal

Critical appraisal was performed to complement and understand the general quality of the evidence retrieved, and therefore, complement its mapping. Due to the variety of designs, the Joanna Briggs Institute guidelines were used (https://jbi.global/critical-appraisal-tools).

### Synthesis of results

The overall characteristics of the studies, including country and type of publication were analysed separately for each sub-question. Findings about the impacts on physical and mental health were summarised by themes. For exposure, the findings were grouped according to exposure factors, geographic areas and group of people exposed. For vulnerabilities, information was also synthesised by groups of vulnerability or susceptibility factors, identifying the main individual or social factors that increase health vulnerability to climate hazards.

The presentation of this ScR follows the recommendations of the Preferred Reporting Items for Systematic reviews and Meta-Analyses extension for Scoping Reviews (PRISMA-ScR).^15^

## Results

A total of 713, 828, 1116, and 931 studies were obtained from all databases combined for physical impacts, mental health impacts, exposures, and vulnerabilities, respectively. Supplementary Tables S1–S4 in the Supplementary Material show the number of references retrieved by each database.

**Fig. 1:**
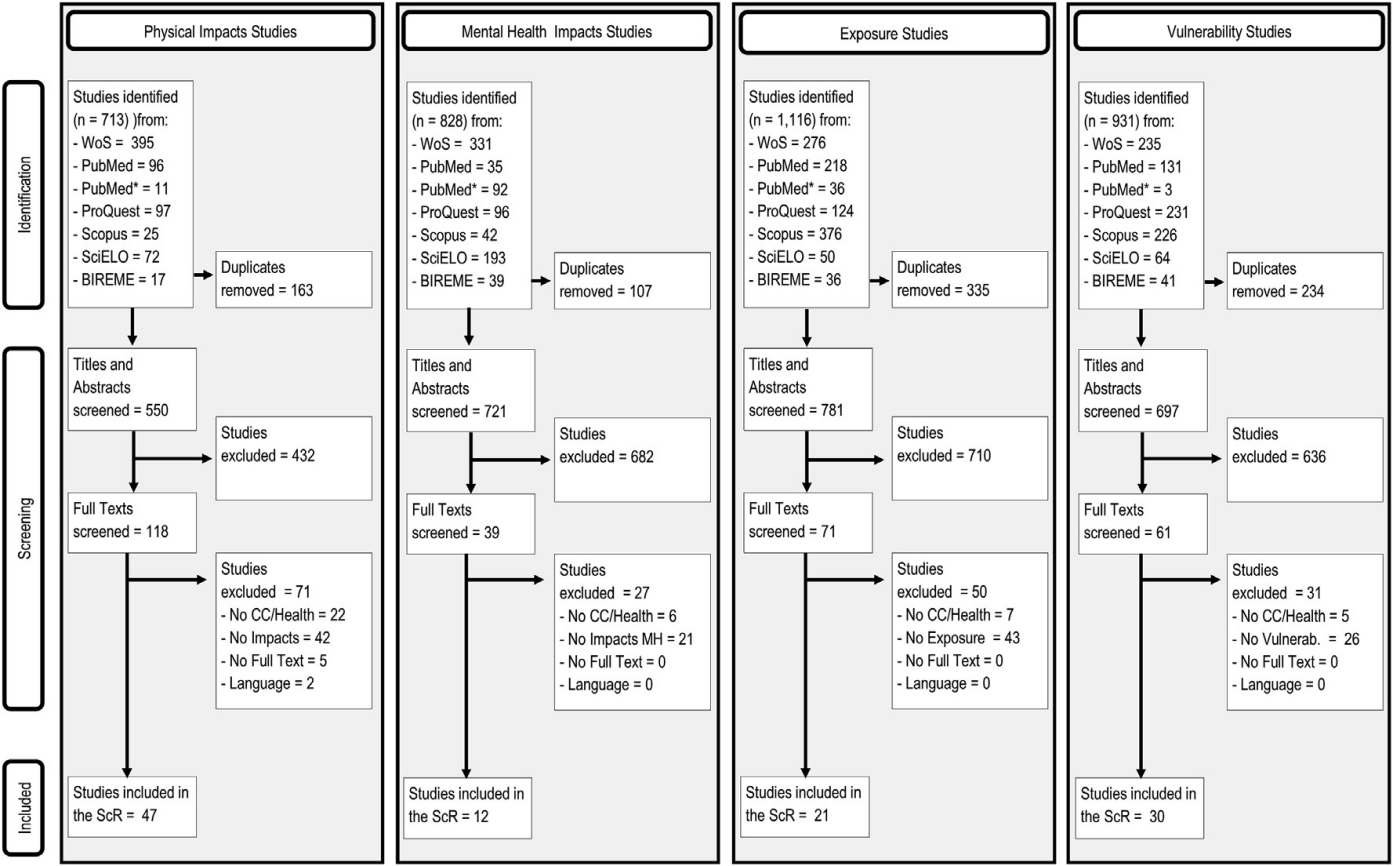
**PRISMA flow diagram of study selection for each sub-question.** WoS: Web of Science; Pubmed∗: PubMed MeSH; ScR: Scoping Review; CC: Climate change; Vulnerab: Vulnerability.

After duplicate removal, eligible studies reduced to 550, 721, 781, and 697 for physical impacts, mental health impacts, exposures, and vulnerabilities, respectively. Fig. 1 shows the studies that were retrieved, screened, selected to be fully analysed, and selected to be included in this ScR.

After screening the title and abstracts as well as full texts, a total of 47 studies were included for physical impacts, 12 for mental health impacts, 21 for exposures, and 30 for vulnerabilities. A detailed analysis of the articles is provided in the following sections.

### Impacts of climate change hazards on physical health and wellbeing in South America

From the 47 full texts included, 39 were focused on a single country, and of those, the majority focused on Brazil (n = 20),^16^, ^17^, ^18^, ^19^, ^20^, ^21^, ^22^, ^23^, ^24^, ^25^, ^26^, ^27^, ^28^, ^29^, ^30^, ^31^, ^32^, ^33^, ^34^, ^35^ followed by Peru (n = 7),^36^, ^37^, ^38^, ^39^, ^40^, ^41^, ^42^ and Colombia (n = 6).^43^, ^44^, ^45^, ^46^, ^47^, ^48^ Table 1 summarises key information of the 47 articles included. Most articles were original studies (n = 37),^17^^,^^18^^,^^20^, ^21^, ^22^, ^23^^,^^25^^,^^26^^,^^28^^,^^29^^,^^31^, ^32^, ^33^, ^34^, ^35^^,^^38^, ^39^, ^40^, ^41^, ^42^, ^43^, ^44^, ^45^, ^46^, ^47^, ^48^^,^^50^, ^51^, ^52^^,^^54^, ^55^, ^56^^,^^58^^,^^62^, ^63^, ^64^, ^65^ primarily time series analyses (n = 28),^17^^,^^18^^,^^20^^,^^22^^,^^23^^,^^25^^,^^26^^,^^28^^,^^31^^,^^33^, ^34^, ^35^^,^^38^^,^^40^^,^^43^, ^44^, ^45^, ^46^, ^47^, ^48^^,^^50^, ^51^, ^52^^,^^56^, ^57^, ^58^^,^^62^^,^^65^ and none of the 10 reviews were systematic reviews.^16^^,^^24^^,^^30^^,^^36^^,^^37^^,^^49^^,^^53^^,^^59^, ^60^, ^61^ Only four studies evaluated the direct impacts of climate change,^26^^,^^27^^,^^32^^,^^59^ and three estimated projections under Representative Concentration Pathways (RCPs) scenarios.^26^^,^^32^^,^^59^ The rest of the studies focused on the association between climatic or meteorological factors and disease frequency.

**Table 1:** Study characteristics, key findings, and overall appraisal of articles on the main impacts of climate change hazards on physical health and wellbeing in South America.

First author	Publication year	Study design	Region or population	Key findings	Overall appraisal
Githeko, A.^49^	2000	Non-systematic review	South America	Malaria increased after heavy rainfall associated with ENSO, which could potentially expand southwards the habitat of *Anopheles darlingi*, it's most efficient vector, potentially doubling the number of people at risk of year-round malaria transmission from 25 to 50 million between 2020 and 2080. Additionally, the high fraction of urban populations coupled with rising temperatures could increase the potential of dengue transmission intensity by a factor of 2–5 and expand southwards, in part due to predominantly urban Aedes mosquitos.	Seek more info
Poveda, G.^42^	2001	Ecological study, time series	Colombia	During non-ENSO years, the malaria regular annual cycle correlates with mean temperature, precipitation, dew point and river discharges. In historical ENSO years (interannual cycles) the timing of the malaria cycle does not change, but cases traditionally increase, correlated to ENSO-related increases in mean temperature and dew point, and decreases in precipitation and river discharges	Seek more info
Cardenas, R.^44^	2006	Ecological study, time series	Colombia	Leshmaniasis incidence increased 7.7–15.7% during El Niño years and decreased 6.8–12.3% during La Niña periods in two provinces in Colombia during 1985–2002. Mean annual leishmaniasis cases between La Niña and El Niño years differed in North Santander and there were marginally significant differences in Santander.	Include
Bell, M.^50^	2008	Case-crossover study	Brazil and Chile	Same and previous day temperature were most strongly associated with lagged mortality risk. Adjustment for ozone or PM10 only lowered the effects but remained positive.	Include
Cardenas, R.^47^	2008	Ecological study, time series	Colombia	Leishmaniasis cases increased 4.98% on average during El Niño years compared to the La Niña years, but with some differences between departments.	Include
Mantilla, G.^46^	2009	Ecological study, time series	Colombia	Change of 1 °C on mean ENSO or ENSO dominant indicator, it’s projected a change of 17.7% or 9.3% the expected number of malaria cases, respectively.	Seek more info
Gutierrez, E.^37^	2010	Ecological study, time series	Peru	A moderate intensity El Niño phenomenon was associated with an increase of medical visits for actinic keratosis, viral warts, and rosacea. La Niña was associated with a reduction in viral warts. Dermatitis and benign neoplasms increased in the spring and summer, respectively. Acne was associated with temperature between 12 and 14 °C.	Seek more info
Romero-Lankao, P.^51^	2013	Ecological study, time series	Colombia and Chile	There was a 0.2% increased risk of cardiovascular deaths associated with a 1 °C higher temperature in the cold season in Bogota. Respiratory and cardiovascular mortality increases were also associated with higher PM10 pollution levels in Bogotá and Santiago in the cold season.	Seek more info
Ferreira, M.^52^	2014	Ecological study, time series	Latin America	Four of the five years with more annual dengue cases in American countries had ENSO activity. The spatial-weighted mean centre of dengue incidence moved approximately 4 °C south in both the northern and southern hemisphere between 1995 and 1998.	Seek more info
Gonzales, G.^36^	2014	Non-systematic review	Peru	Climate change may impact the frequency and severity of ENSO, which in Per has been associated with increased incidence of cholera, diarrheal disease, malaria and dengue. Climate change also leads to higher temperatures that may further expand the endemic areas of some vector-borne diseases, and magnify air pollution leading to increased respiratory and cardiovascular disease	Seek more info
Pan, W.^53^	2014	Non-systematic review	Amazon	The 2012 floods in Loreto, Peru, probably increased malaria cases in at least three ways: 1) the excess rainfall and high temperatures extended the transmission season, 2) flooding displaced families into closer proximity of anopheline vectors for prolonged periods, and 3) flood response efforts took away energy from malaria prevention efforts. Finally, the 2010 drought and increased temperatures could have accelerated Plasmodium’s development in Anopheles mosquitoes.	Include
Sena, A.^23^	2014	Non-systematic review	Brazil	In Brazil, the semiarid northeast is a historically and permanently dry area, where extreme drought occurs periodically affecting a large population and causing population displacement and economic losses. The northeast also has some of the worst health and wellbeing indicators in Brazil. Although social and economic vulnerabilities have been partially reduced, climate change will probably impact severely this region.	Include
Smith, L.^17^	2014	Ecological study, time series	Brazil	A 1.3%–181% increase in hospitalisations for respiratory diseases in children under-five was observed in the 77 (31.3%) municipalities highly exposed to drought, compared to their 10-year mean. A 1.2%–267% increase was observed in 197 (43.0%) municipalities affected by the 2010 drought. Aerosol was the main factor associated with hospitalisations in drought-affected municipalities during 2005, and human development conditions may have mitigated the impacts in 2010	Include
Warner, K.^41^	2014	Mixed methods study	Peru	Two-thirds of Huancayo households sustained crop damage and lower crop yields, 42% experience substantial negative impacts on household income from rainfall variability. Rainfall changes affected the ability of households to feed themselves and earn livelihoods, with over 80% of households responding to survey that they experienced decreases in harvest, livestock, and own food consumption in the past 5–10 years.	Seek more info
Quintero-Herrera, L.^45^	2015	Ecological study, time series	Colombia	Mean dengue cases differed significantly during El Niño, the climate neutral period, and La Niña. The dengue incidence rate was independently associated to both Oceanic Niño Index and pluviometry, after adjusting for year and week.	Seek more info
Son, J.^33^	2016	Ecological study, time series	Brazil	Cold temperatures have larger effects on mortality than temperatures in subtropical cities, acting primarily via cardiovascular and respiratory mortality, respectively. Risks were higher for females and people with no education about heat effects, and males for cold effects. Older persons and widows had higher mortality risks for heat and cold. Mortality during heat waves was higher than on non-heat wave days for total, cardiovascular, and respiratory mortality. Both heat and cold effects remained after adjusting for pollution markers (PM10 and ozone)	Include
Duarte, J.^30^	2017	Ecological study, time series	Brazil	There is a significant positive association between the Acre river level and hospitalisation rates due to diarrhoea, with a 7% increase in the diarrhoea rates for each meter of river level increase.	Include
Nava, A.^29^	2017	Non-systematic review	Brazil	Rapidly urbanizing and deforested rural areas present an increased risk for leptospirosis via floods, dengue, Chikungunya and Zika, hantavirus outbreaks, and yellow fever. Increased incidence of urban arboviruses is associated with areas with more frequent rainfall and severe droughts, since both factors can favour breeding sites for the *Aedes* spp. and *Culex* spp vectors.	Include
Peña-García, V.^43^	2017	Ecological study, time series	Colombia	Cities with highest temperatures had negative correlations between temperature and dengue incidence while cities with lower temperatures had positive correlations. Weekly dengue incidence had an inverted U-shaped association with lower, minimum and maximum mean temperature.	Seek more info
da Costa, S.^19^	2018	Ecological study, time series	Brazil	Niche modelling identified that the preferential habitat of *Lu. whitmani* includes annual precipitation between 1000 and 1600 mm, intermediate vegetation density, 15–21 °C average temperature of the coldest quarter, and 19–24 °C annual average temperature. American cutaneous leishmaniasis is associated to areas of intermediate vegetation, rainfall of 800–1200 mm, coldest quarter mean temperature >16 °C and annual mean temperature <23 °C.	Include
Leal, W.^15^	2018	Non-systematic review	Brazil	Weather pattern shifts can also favour new vector niches. Such changes could have influenced the recent Zika and Chikungunya emergencies in Brazil, amplified by one of the strongest El Nino event in recent years. There is limited local evidence to understand these patterns and use and support disease control.	Seek more info
Tapia-Garay, V.^48^	2018	Ecological study	Chile	The maximum temperature in the warmest month and precipitations in the driest month correlated importantly with the distribution of Chagas’ disease and *T. infestans* in Chile. Annual precipitation, temperature seasonality and average temperature additionally contributed to Chagas’ disease distribution.	Include
Laneri, K.^54^	2019	Ecological study, time series	Argentina	There were lagged, non-linear correlations between malaria cases and maximum and minimum temperature and humidity.	Seek more info
Lopes de Moraes, S.^24^	2019	Ecological study, time series	Brazil	Childhood hospitalisations for respiratory diseases were statistically significantly higher for mean air temperature (17.5–21 °C), relative air humidity (84%–98% for females only), precipitation (0–2.3 mm for total and both sexes and >120 mm for females) and PM10 (>35 μg/m^3^ for total and females).	Seek more info
Oliveira, V.^55^	2019	Ecological study, time series	Brazil	There is a negative correlation between rainfall levels and childhood mortality rate. Exposure to infectious diseases may be the main potential mechanism. Crude mortality rate correlates strongly with episodes of severe/extreme droughts, events that will become more frequent and intense in the Brazilian Northeast region under current climate change predictions. Estimates indicate that the childhood human capital loss due to rainfall reductions can reach 1.5% of the state GDP by year 2100	Include
Silveira, I.^28^	2019	Ecological study	Brazil	Higher cardiovascular mortality was associated with the lowest and highest temperatures in Brazil overall and the regions, presenting a U-shaped exposure- response relationship in most cities. The minimum mortality temperature was 27.7 °C (79th percentile), ranging from 25.2 °C (85th percentile) in the South region to 29.6 °C (90th percentile) in the North region.	Include
Colston, J.^40^	2020	Cohort study	Peru	In the early flood period (Dec01-Feb29) when the study community was flooded and many families were displaced there was increased risk of heat-stable enterotoxigenic *E. coli* and decreased risk of enteric adenovirus. In the later flood period (Mar01-May31) when evacuees returned to their communities, but rains and flooding continued, there was sharply increased risk of rotavirus and sapovirus, and increased *Shigella* spp. transmission and *Campylobacter* spp.	Include
da Silva Neto, A.^34^	2020	Ecological study, time series	Brazil	The annual incidence of visceral leishmaniasis in Mato Grosso do Sul from 2002 to 2015 correlated negatively with both the mean 3.4 El Niño index variation and soil moisture.	Include
Deshpande, A.^56^	2020	Ecological study, time series	Ecuador	In rural areas, there were no significant associations between heavy rainfall events and lagged diarrhoea incidence. In urban areas, however, dry antecedent conditions were associated with higher incidence than wet antecedents. Also, heavy rainfall events with dry antecedent conditions were associated with 35% higher incidence compared with similar conditions without heavy rainfall events.	Include
Ellwanger, J.^57^	2020	Non-systematic review	Amazon Basin	Amazon deforestation is a key and well-known driver of climate change through different mechanisms. Through an array of direct and indirect mechanisms, deforestation in the Amazon has an important impact in the risk of infectious diseases and public health. Mitigation is critically needed to address this threat	Include
Ferro, I.^58^	2020	Ecological study, time series	Argentina	Two abrupt increases in hantavirus pulmonary syndrome (HPS) cases were observed in north-western Argentina between 1997 and 2017: 1) spring 2006 to autumn 2007, and 2) summer and autumn 2015. Rainfall and temperature lagged 2–6 months correlated with HPS incidence. A biannual model with rainfall and temperature in the past 6 months (1 lagged period) explained 69% of the variation in HPS cases.	Seek more info
Geirinhas, J.^21^	2020	Ecological study, time series	Brazil	There were four extremely intense heatwaves between 2010 and 2012. The highest absolute mortality during heat-related events was related to cardiovascular illnesses but the highest mortality excess was diabetes-related, particularly among elderly women. Cumulative heat stress over consecutive days, especially if preceded by persistent high temperature periods, led to higher excess mortality rather than sporadic, single hot days	Seek more info
Lowe, R.^59^	2020	Non-systematic review	Amazon Basin	The Amazon is one of the richest reservoirs of arboviruses in the world and in addition has seen the expansion of dengue and the introduction of Chikungunya and Zika into their growing urban areas, posing a major threat. The advance of deforestation and land-use change in the Amazon against a backdrop of climate change is possibly approaching an irreversible tipping point to becoming a degraded savanna-like ecosystem. This would harm the global climate system and would lead to increased droughts and fires, warmer temperatures and weather anomalies among many other consequences that would favour the transmission of arbovirus as it has been seen with the impacts of ENSO.	Include
Machado-Silva, F.^16^	2020	Ecological study, time series	Brazil	There was an increase in respiratory-disease hospitalisation in drought years but also a decrease in asthma cases, both possibly due to lower rainfall. There was also an increase in respiratory hospitalisations in the fire season, possibly due to smoke production.	Include
Palmeiro-Silva, Y.^60^	2020	Non-systematic review	Chile	There is limited evidence in Chile regarding the impact of climate change on health. High temperatures were associated with higher mortality risk in the elderly and there is greater exposure to heatwaves.	Seek more info
Thoisy, B.^27^	2020	Ecological study, time series	Brazil	Models of human cases of Yellow Fever in 2017 include precipitation seasonality, temperature seasonality, precipitation of warmest quarter, precipitation of driest month, human footprint index, urban expansion, mammal richness, and vaccination coverage. The Amazon basin overall primarily remains at lower risk and the most favourable conditions in both projections remain focused in surrounding forest regions and potentially the northwest.	Seek more info
Ambikapati, R.^38^	2021	Cohort study	Peru	Plantain and yucca prices increased after the two La Niña periods, some of these increases concurrently with drops in child consumption. After adjusting for covariates, the frequency of grains, rice, dairy and sugar in meals fell by 5–7% but plantains’ frequency in meals increased 24%. There were lower intakes of yucca and rice (7 and 3 g) during ENSO exposure but inconsistent across different ENSO indices. Girls consumed 10–12 g less sugar than boys during ENSO. Overall, ENSO phases did not affect dietary diversity (DD) but the severity of ENSO had varied effects on DD.	Include
Butt, E.^22^	2021	Ecological study, time series	Brazil	Deforestation growth since 2012 increased the dry season fire count in 2019 by 39%, potentially resulting in 3400 additional deaths in 2019 to increased particulate air pollution. If deforestation in 2019 had reached the maximum recorded in 2003–2019, active fire counts would have doubled leading to 7900 additional premature deaths. The prevention of all fires in the Brazilian Amazon would have avoided 367,429 DALYs and 9469 premature deaths in 2019.	Seek further info
de Souza, A.^61^	2021	Ecological study, time series	Brazil	Dengue incidence in 2008–2018 was associated with minimum temperature or proportion of days with temperature >26 °C lagged one month.	Include
Delahoy, M.^39^	2021	Ecological study, time series	Peru	A 1 °C increase in temperature is associated with 3.8% more childhood diarrhoea clinic visits three weeks later. Adjusting for temperature, there was a higher incidence rate of childhood diarrhoea clinic visits during moderate/strong El Niño events and during the dry season. There was no evidence that access to piped water mitigated the effects of temperature on diarrhoea incidence.	Include
Gracie, R.^20^	2021	Ecological study	Brazil	Leptospirosis incidence was higher in municipalities with floods in all five population-size municipalities and also in municipalities experiencing more outbreaks. Regression trees showed that the fraction of households with pit sewage disposal, >3 flood events, and cities in the second level of population size had different leptospirosis incidence.	Include
Hamlet, A.^62^	2021	Ecological study, time series	South America	The interannual and seasonal models reproduced well the spatiotemporal heterogeneities in Yellow Fever cases. The primary contributors of the interannual model were EVI, land-cover and vegetation heterogeneity, perhaps a proxy for habitat fragmentation, and for the seasonal model EVI, day temperature and rainfall amplitude.	Seek more info
Højgaard Borg, F.^35^	2021	Non-systematic review	Peru	Participants screened after a flood reported a 10% depression prevalence and 36% domestic violence, and half of the participants accepted help and accompaniment by public health services after screening.	Seek more info
Olmos, M.^63^	2021	Case report	Ecuador	Reported dengue cases in 2015, an ENSO year with an extended the season of warm weather and precipitation that could have made the environment more suitable for mosquito expansion, more than tripled cases in 2011–2014	Include
Sadeghieh, T.^31^	2021	Ecological study	Brazil	The increases in Zika are due to more favourable climate for mosquitoes, with the mean temperature reaching 28 °C in the warmest months.	Seek more info
Silveira, I.^25^	2021	Ecological study, time series	Brazil	There was a trend of lower mortality related to low temperatures but higher mortality related to high temperatures in all the models and scenarios evaluated. In most cities there were net increases in the excess temperature-related mortality, with larger increases in the higher emission scenario, RCP8.5 and the Eta-HadGEM2-ES model. The RCP8.5 projections estimate that the temperature-related mortality fractions in 2090–2099 compared to 2010–2019 would increase by 8.6% and 1.7%, under Eta-HadGEM2-ES and Eta-MIROC5, respectively, and 0.7% and −0.6% under RCP4.5	Seek more info
Soares da Silva, A.^64^	2021	Ecological study, time series	Brazil	La Niña events increased rainfall levels or early rains in November and led to increased leishmaniasis from January to March. An inverse effect is observed in El Niño years, when most leishmaniasis cases occur later, from March to May.	Include

ENSO = El Niño–Southern oscillation; DALYs = disability-adjusted life years; HPS = hantavirus pulmonary syndrome; EVI = enhanced vegetation index; PM = particulate matter; GDP = gross domestic product.

The 37 original studies were scattered among multiple topics, including mortality (n = 6),^22^^,^^26^^,^^27^^,^^34^^,^^52^^,^^64^ dengue fever (n = 5),^33^^,^^44^^,^^46^^,^^55^^,^^58^ leishmaniasis (n = 5),^20^^,^^35^^,^^45^^,^^48^^,^^65^ diarrheal disease (n = 4),^31^^,^^40^^,^^41^^,^^62^ malaria (n = 3),^43^^,^^47^^,^^51^ and respiratory diseases (n = 3).^17^^,^^18^^,^^25^ The main study covariates were primarily climate-related (n = 23) and El Niño–Southern Oscillation (ENSO) (n = 12),^35^^,^^38^, ^39^, ^40^^,^^43^^,^^45^, ^46^, ^47^, ^48^^,^^55^^,^^58^^,^^65^ but also extreme weather events, including extreme temperatures and floods (n = 5),^21^^,^^22^^,^^34^^,^^41^^,^^64^ emission levels and air pollution (n = 5),^25^^,^^29^^,^^32^^,^^34^^,^^52^ vegetation/deforestation (n = 3),^20^^,^^23^^,^^50^ and droughts or fire (n = 3).^20^^,^^23^^,^^50^

### Impacts of climate change hazards on mental health and wellbeing in south America

Twelve studies were included in relation to mental health and wellbeing impacts, and most of them presented findings from Brazil (n = 4)^24^^,^^66^, ^67^, ^68^ and Peru (n = 3).^69^, ^70^, ^71^ The articles primarily identified floods, droughts, and ambient temperature as climate-related hazards that affect mental health and wellbeing. The most studied mental health outcomes were common mental disorders, including trauma, depression, and anxiety,^24^^,^^70^^,^^71^ and domestic violence.^69^^,^^71^ Table 2 summarises the main characteristics and overall appraisal of the articles.

**Table 2:** Study characteristics, key findings, and overall appraisal of articles on the main impacts of climate change hazards on mental health and wellbeing in South America.

First author	Publication year	Study design	Region or population	Key findings	Overall appraisal
Team V.^72^	2011	Non-systematic review	Argentina, Australia, Chile, New Zealand, and South Africa.	Climate change particularly impacts on water and food security, extreme weather events and migration. Projections indicate that the continuing impact of climate change may precipitate political and socioeconomic crises, including increased local, regional, and international migration.	Include
Sena, A.^23^	2014	Non-systematic literature review	Brazil	In Brazil, 50% of disaster events are drought-related and the semiarid northeast region of Brazil is especially affected This region has worse health and well-being indicators than the rest of the country: stress, anxiety, depression, behavioural changes, and violence.	Include
Espinoza-Neyra, C.^70^	2017	Cross-sectional study	Residents of Rio Seco, Trujillo (Peru) exposed 6 weeks before to El Niño Costero floods	PTSD: 89 screened cases (PR 48.4% 95% CI: 40.9–55.9). Cases were more frequent amongst separated persons, with a monthly income of >500 PEN (USD 106) with a destroyed or uninhabitable home. Having an income between 500 and 1000 PEN (USD 107–257) decreased the odds of having PTSD in 45%.	Include
Manfrini G.^68^	2017	Qualitative study	Rural area of Santa Catarina State, Southern Brazil.	Losses associated with disasters influenced social lives, daily routines and the preservation of cultural values. The interviewed families made their interpretations based on their life experiences and by comparing and evaluating the event’s impact in relation to other affected families. Their perception of the event’s magnitude tended to focus on damage and loss, reducing their understanding of the context of the event to a micro level, without any association with prior knowledge of the risks to which they were exposed. Their narratives denoted the family vulnerability and resilience in relation to the unexpected disaster transition, and the post-disaster recovery of the family in its social, economic, and environmental dimensions.	Include
Sapiains, R.^73^	2017	Non-systematic literature review	Chile and Latin America	Case studies conducted in Chile illustrate the findings. Some relate to vulnerability, and stress due to the perceived effects of climate change and there is a need to dialogue between the holders of traditional knowledges and scientists, to achieve a joint work with the communities on mitigation and adaptation strategies.	Include
Sapiains, R.^74^	2017	Non-systematic literature review	Chile and Latin America	Psychological aspects of climate change in Latin America focused on social vulnerability, inequalities, poverty alleviation, political participation and agriculture in rural and indigenous communities in the context of droughts or water management related issues.	Include
Seixas S.^75^	2017	Literature Review	Worldwide	An understanding of mental health emerges in relation to processes of environmental change. Most of the examined studies have dealt with the effects of temperature and major external events (e.g., droughts, floods, cyclones and storms) on physical and mental health, and the subsequent problems they instigate (e.g., the degradation of ecosystems services and the eradication of livelihoods). Most studies have examined social groups that have suffered directly from the traumas caused by such events, with particular consideration for the degree of vulnerability to which these social groups are exposed.	Include
Akpinar-Elci M.^76^	2018	Cross-sectional study	Guyana	Individuals whose homes had flooded previously had slightly more risk for experiencing diminished interest in daily activities, diminished involvement in social activities, and an increased difficulty in concentrating.	Include
Contreras, C.^71^	2018	Case report	Informal settlements in the urban outskirts of Lima	Screening for 2 outcomes across two sites: Depression (PHQ-9) 12/116 (10%) cases were identified and Domestic Violence (MCVQ) 21/58 (36%) were cases identified.	Include
Kim Y.^67^	2019	Time-series meta-regression	Brazil	Overall, higher ambient temperature was associated with an increased risk of suicide. Brazil had an unclear association, with the highest risk at 24.8 °C. The RR 1.15 (95% CI: 1.02, 1.30) compared with the risk at the first percentile.	Include
Da Silva, L.^66^	2020	Time-series study	Curitiba, Brazil	There were significant associations between environmental conditions (10 μg/m^3^ increase in air pollutants and temperature °C) and hospitalisations by mental behavioural disorders. The air temperature had the highest relative risk at 0-day lag. Ozone exposure was a risk for women, higher for younger age group. Elders from both sexes were more susceptible to temperature variability.	Include
Loayza-Alarico, M.^69^	2021	Case control study	Displaced families in shelters of Castilla, Catacaos, Cura Mori, Narihualá and Simbilá in Piura.	At 3- and 9-months assessments, the families presented significant differences in health disorders and family violence. 26.5% had some psychological disorder associated with living in shelters. Families with less than 80% safe water management and safe water storage had higher risks of acute diarrheal diseases.	Include

PTSD = post traumatic stress disorder; OR = odds ratio; RR = relative risk.

Five non-systematic literature reviews^24^^,^^72^^,^^73^^,^^75^ described evidence regarding social and economic vulnerability stressors, climate change perceptions, adaptive capacity, and inequalities experienced by groups living in high-risk settings for climatic events. All these reviews highlighted the small number of available studies from Latin America that assessed mental health outcomes. Two cross-sectional studies conducted in Peru^70^ and Guyana,^76^ one longitudinal case-control study,^69^ and one case report conducted in Peru explored flood-related impacts and mental disorders.^71^ Two time-series studies explored the relationship between environmental stressors (heat, humidity, air pollutants) and hospitalisation rates and suicide cases.^66^^,^^67^ And there was a qualitative case study exploring family transitions and the impact of an unexpected disaster.^68^

The critical appraisal concluded that all identified articles should be included in the review.

### Exposure of south American populations to climate change hazards

A total of 21 articles related to population exposure to climate change hazards in South America were identified, covering Brazil (n = 9),^20^^,^^74^^,^^77^, ^78^, ^79^, ^80^, ^81^, ^82^, ^83^ Ecuador (n = 3),^84^, ^85^, ^86^ Peru (n = 2),^37^^,^^87^ Chile (n = 1),^61^ and Argentina (n = 1).^88^ The rest of the articles included SA countries within global assessments (n = 5).^89^, ^90^, ^91^, ^92^, ^93^ Among the environmental factors that people have been exposed to, those that have been studied in SA include heat exposure (n = 11),^61^^,^^74^^,^^77^, ^78^, ^79^, ^80^, ^81^^,^^83^^,^^89^^,^^91^^,^^93^ exposure to several pathogens, especially those associated with vector-borne diseases (n = 6),^20^^,^^37^^,^^85^, ^86^, ^87^^,^^94^ air pollution (n = 2),^88^^,^^90^ droughts (n = 1),^95^ and UV radiation (n = 1).^84^ The countries and regions identified as those with the greatest exposure are the tropics and subtropics,^91^ with the Amazon in Brazil and Ecuador being the most studied.^74^^,^^77^, ^78^, ^79^, ^80^ Brazil is one of the countries with the largest crude population exposed to 30 °C and above wet-bulb temperature,^93^ with the central west, the northeast, and southeast regions being the most affected.^74^^,^^78^^,^^80^^,^^83^

As a social group, the sugarcane cutter workers from the coast of Ecuador were identified as highly exposed to heat stress and UV radiation.^89^ Additionally, due to ecological changes driven by climate change added to natural variations of the climate (e.g., ENSO), general population have been greatly exposed to larger droughts and more intense precipitation events.^37^ These changes increase the exposure to water- and vector-borne diseases by favouring the conditions to microorganisms or changing the geographical niches towards, for example, mountainous areas.^20^^,^^37^^,^^85^^,^^86^ Table 3 shows a summary of the articles included in this section.

**Table 3:** Study characteristics, key findings, and overall appraisal of articles on to what extent human populations in South America are exposed to the hazards of climate change.

First author	Publication year	Study design	Region or population	Key findings	Overall appraisal
Gonzales, G.^36^	2014	Non-systematic review	Peru	Urban infestation of Chagas disease vectors were reported in Arequipa, Peru and a variable that can explain this is the rise of temperature. ENSO was associated with a proliferation of *V. cholerae* in epidemics due to the rise of temperature.	Seek further info
Carreras, H.^89^	2015	Ecological study	Córdoba, Argentina	People exposed to air pollution (PM10) were significantly affected by daily temperature range, increasing the risk of hospital admissions.	Seek further info
Brondízio, E.^95^	2016	Non-systematic review	Amazon Basin	The highest incidence of diarrheal diseases appears to occur in the rainy season and cities most affected are the ones with poor sanitation systems.	Seek further info
Escobar, L.^86^	2016	Ecological study	Ecuador	The populations in the Andean highlands (Ecuadorian regions) would be increasingly exposed to disease vectors as the future climate changes unfold due to likely upward vector species range shifts.	Include
Glaser, J.^90^	2016	Non-systematic review	Worldwide	People exposed to warmer temperatures, coupled with decreasing precipitation, might exacerbate this kidney diseases by reducing water supply and water quality.	Seek further info
Harari, R.^85^	2016	Non-systematic review	Ecuador	In the Ecuadorian coast, sugarcane cutters are exposed to high ambient temperatures and poor working conditions. This occupational group has a high prevalence of kidney diseases and skin cancer.	Seek further info
Guo, Y.^92^	2018	Ecological study	Worldwide	The communities close to the equator or located in tropical or subtropical climates are projected to have a large increase relative risks of mortality associated with heatwaves, and those located in temperate regions are projected to experience a relatively small increase.	Include
Miranda da Costa, S.^19^	2018	Ecological study	Brazil	Changes in the environment might lead to an expansion of the Lutzomyia (Nyssomyia) whitmani in the northern region, especially the State of Amazonas.	Seek further info
Zhao, Q.^80^	2018	Ecological study	Brazil	People exposed to high temperature variability have greater risk of hospitalisations, especially due to respiratory causes.	Seek further info
Corrêa, M.P.^91^	2019	Ecological study	South America and Antarctica	Exposure of people to UV radiation differ by latitude.	Seek further info
Lippi, C.^87^	2019	Ecological study	Ecuador	The human population with the potential to experience increased exposure to mosquito presence generally increases with RCP. *Ae. aegypti* would expand into mountainous areas, exposing people living in transitional areas to vector-borne diseases.	Include
Wang, F.^94^	2019	Ecological study	Worldwide	People exposure to wet bulb temperature above 30–32 °C would increase significantly in the middle- and low-latitude regions.	Seek further info
Zhao, Q.^77^	2019	Ecological study	Brazil	Exposure to ambient heat was positively associated with hospitalisation for COPD, particularly during the late hot season. The effect on heat was greater in regions like the central west and southeast and minimal in the northeast.	Seek further info
Zhao, Q.^78^	2019	Ecological study	Brazil	Risk of hospitalization associated with heat exposure was greater for children aged 9 or younger and for people aged 80 or older than for middle-aged adults.	Seek further info
Zhao, Q.^79^	2019	Ecological study	Brazil	Exposure to hot seasons increase the risk of hospitalisations, especially among children and adults above 60 years old.	Seek further info
Zhao, Q.^81^	2019	Ecological study	Brazil	People exposed to heatwaves have greater risk of hospitalisations, especially due to endocrine, nutritional and metabolic diseases, skin problems, and genitourinary diseases.	Seek further info
Charette, M.^88^	2020	Ecological study	Peruvian Amazon	Exposed children and people older than 65 years old increase the risk of dengue. The effect of temperature on dengue depended on season, with stronger effects during rainy seasons.	Include
Liu, Y.^96^	2020	Ecological study	Worldwide	Among the climate change scenarios SSP, the highest population exposure to droughts is likely under the SSP3 scenario in 2046–2065, with a 62% increase compared with that in the base period, whereas the lowest exposure was likely under the SSP1 scenario in 2016–2035, with a 30% increase compared with that in the base period.	Seek further info
Alves de Oliveira, B.^82^	2021	Ecological study	Brazilian Amazon	Heat stress exposure due to deforestation was comparable to the effect of climate change under RCP8.5. By 2100, savannisation of the Amazon will lead to more than 11 million people being exposed to heat stress.	Include
Jacobson, L.^84^	2021	Ecological study	Brazil	People exposed to extreme cold or extreme heat changed depending on the geographical location, but both are associated with higher mortality.	Include
Palmeiro-Silva, Y.^60^	2020	Non-systematic review	Chile	The number of heatwave exposures for the elderly has increased over time. Wildfire exposure has almost tripled when comparing the periods 2011–2004 and 2015–2018.	Seek further info

RCP = representative concentration pathway; PM = particulate matter; SSP = shared socioeconomic pathway; ENSO = El Niño-Southern Oscillation; COPD = chronic obstructive pulmonary disease.

### Vulnerabilities or susceptibility factors present in south American populations

A total of 30 articles identified vulnerabilities or susceptibility factors that might increase or decrease the risk of negative impacts of climate hazards on health and wellbeing of people in SA. The majority were from Brazil (n = 14),^22^^,^^31^^,^^96^, ^97^, ^98^, ^99^, ^100^, ^101^, ^102^, ^103^, ^104^, ^105^, ^106^, ^107^ six were from Peru,^40^^,^^87^^,^^108^, ^109^, ^110^, ^111^ five had a worldwide scope,^87^^,^^112^, ^113^, ^114^, ^115^ three covered Latin America,^64^^,^^116^^,^^117^ and two were from Ecuador.^118^^,^^119^ The articles were published between 2005 and 2021, with most of them being ecological studies,^22^^,^^31^^,^^40^^,^^87^^,^^95^, ^96^, ^97^, ^98^^,^^100^, ^101^, ^102^, ^103^, ^104^, ^105^, ^106^, ^107^, ^108^, ^109^^,^^112^, ^113^, ^114^, ^115^^,^^117^ followed by case studies,^110^^,^^119^ qualitative studies,^111^^,^^118^ cross-over studies,^64^ and reviews.^116^ Table 4 shows a summary of the articles included and their overall critical appraisal.

**Table 4:** Study characteristics, key findings, and overall appraisal of articles on main vulnerabilities or susceptibility factors present in the South American human population groups that could increase the risk of climate change adverse impacts on health and wellbeing (n = 30).

First author	Publication year	Study design	Region or population	Key findings	Overall appraisal
Carey, M.^111^	2005	Case report	Cordillera Blanca, Perú	Vulnerability factors to avalanches and floods: i) poor communication between local people, scientists, and policymakers; ii) unstable economy and governmental institutions.	Include
Bell, M.^50^	2008	Cross-over study	Brazil, Chile, and Mexico	Susceptibility factors: age, gender, educational status.	Include
Sullivan, C.A.^116^	2009	Ecological study	Worldwide	Vulnerability factors: i) property rights and access, relatively lower and less reliable resource assets; ii) lower degree of human and institutional capacity, with a higher geospatial risk.	Include
Mark, B.^110^	2010	Ecological study	Cordillera Blanca, Peru	Vulnerability factors: governance and conflicts that affect household access to key livelihood resources such as land and water.	Include
Samson, J.^113^	2011	Ecological study	Worldwide	Vulnerability factor: regions with high population density have higher risk of climate impacts in comparison to regions with low population density.	Seek further info
De Oliveira, T.^99^	2012	Ecological study	Rio de Janeiro, Brazil	Vulnerability factors: i) high density population; ii) sanitation and garbage problems increase risk to leptospirosis; iii) overcrowding and lack of plan.	Seek further info
Confalonieri, U.	2014	Ecological study	Brazil	Vulnerability indicator based on population projections; climate-induced migration scenarios; disease trends; desertification rates; economic projections (GDP and employment) and projections for health care costs.	Include
Guimaraez^101^	2014	Ecological study	Brazil	Vulnerability index “Índice de Vulnerabilidade Socioambiental” based on: age, gender, rural/urban areas, human development index, Gini index, urban development.	Include
Barbieri, A.^104^	2015	Ecological study	Minas Gerais, Brazil	Vulnerability factors: combination of i) health population status; ii) economy (household consumption, gross regional product, employment); iii) institutional capacity (municipal contingency plan to manage hazards), and iv) demographic (age composition, households with access to proper sanitation, and expected years of education) dimensions.	Include
Qin, H.^118^	2015	Ecological study	Bogotá, Colombia; Buenos Aires, Argentina; Mexico City, Mexico; and Santiago, Chile	Vulnerability factors: i) community communication and interaction have a strong effect on how urban populations respond to climate hazards; ii) access to information sources about environmental emergencies was significantly related to household members’ receiving emergency support and public healthcare.	Include
Debortoli, N.^97^	2017	Ecological study	Brazil	Main vulnerability factor: steep slopes or degraded/deforested areas under extreme rainfall events.	Include
Quintão, A.^106^	2017	Ecological study	Brazil	Vulnerability indicator “Index of Human Vulnerability” based on: i) indicator of vegetation cover, natural disasters, and health; ii) indicator of poverty and a sociodemographic index; iii) index of municipality development and primary health care coverage; iii) climate index.	Include
Sena, A.^103^	2017	Ecological study	Municipalities in and outside the semiarid region of Brazil.	Vulnerability factors to droughts at municipal level: i) access to piped water; ii) illiteracy and poverty; iii) the living conditions.	Include
Sorensen, C.^120^	2017	Case report	Manabí, Ecuador	Vulnerability factors: i) capacity to respond, adapt, and recover; ii) resources; iii) lack of cohesion; iv) poor housing conditions; and v) inadequate access to piped water in the home.	Include
Nagy, G.^117^	2018	Review and survey	Latin America	Vulnerability factors: i) socioeconomic determinants of human wellbeing and health inequalities; ii) lack of public awareness, investment, and preparedness.	Include
Zavaleta, C.^112^	2018	Qualitative study	Peru	Vulnerability factors: i) demographic drivers (a growing population); ii) natural resource degradation (deforestation) coupled with limited opportunities to increase incomes.	Seek further info
dos Santos, RB^100^	2019	Ecological study	Espírito Santo, Brazil	Vulnerability factors: sociodemographic (e.g., income, political organisation), economic (e.g., poverty), and environmental characteristics (e.g., vegetation cover).	Include
Duarte, J.^30^	2019	Ecological study	Rio Branco, Brazil	Susceptibility factors: Age. In this case, the most affected group was children less than one year old.	Seek further info
Lapola, D.^108^	2019	Ecological study	Manaus, Natal, Vitória, São Paulo, Curitiba, and Porto Alegre, Brazil	Vulnerability index calculated based on distribution of >65-year-old elderly people, human development index, and temperature.	Include
Lee, J.^115^	2019	Ecological study	Worldwide	Susceptibility factors: obese and elderly population proportion. Other vulnerability factors: total health expenditure per capita.	Seek further info
Ramirez, I.^109^	2019	Ecological study	Peru	Susceptibility factors: i) pre-existing socioeconomic status (e.g., poverty), and ii) health, infrastructure, and gender conditions.	Include
Tauzer, E.^119^	2019	Qualitative study	Periurban areas Machala, Ecuador	i) Susceptible population identified: children, elderly people, physically disabled people, low-income families, and recent migrants. ii) Other vulnerability factors for floodings: blocked drainage areas, overflowing canals, collapsed sewer systems, low local elevation, weak adaptive capacity due to lack of social organisation, weak political engagement and financial capital, and general forecasts.	Include
Vommaro, F.^102^	2019	Ecological study	Maranhão, Brazil	Main vulnerability factor: adaptive capacity. Other factors: poverty and socio-demographic development.	Include
Chambers, J.^114^	2020	Ecological study	Worldwide	Susceptibility factor: age. Other vulnerability factors: socioeconomic status at country level and country health system capacity. Certain countries are at additional risk of negative impacts due to the combination of high heatwave exposure, low medical staffing, and low income.	Include
Charette, M.^88^	2020	Ecological study	Pucallpa, Peruvian Amazon	Susceptibility factors: age and gender. Young child or elderly, being female.	Include
Geirinhas, J.^21^	2020	Ecological study	Metropolitan Region of Rio de Janeiro	Vulnerability factors to mortality due to temperature extremes: people with diabetes, particularly for women within the elderly age groups.	Include
Liu, Y.^96^	2020	Ecological study	Worldwide	Vulnerability factors to droughts: socioeconomic (gross domestic product); agricultural (fraction of cropland); infrastructure (total water withdrawal per capita/total renewable water resources per capita).	Seek further info
Xu, R.^107^	2020	Ecological study	Brazil	Susceptibility factors: age, socioeconomic status, pre-existing health conditions. Other vulnerability factors: socioeconomic disparities.	Include
Delahoy, M.^39^	2021	Ecological study	Peru	Vulnerability factors: access to piped water (positive association)	Seek further info
Menezes, J.^98^	2021	Ecological study	Brazil	Vulnerability factors to drought: social determinants (human welfare, economic development, income, education, quality of life), rural characteristics, access to water, and social inequality.	Include

GDP = gross domestic product.

From the evidence it is possible to identify four groups of vulnerability or susceptibility factors: *i)* individual factors; *ii*) geographical features of the natural or built environment; *iii*) general social determinants of health; and *iv*) wider policy and institutional capacities. Among individual susceptibility factors, the following were identified: life-stage or age,^64^^,^^87^^,^^100^^,^^106^^,^^113^ particularly young^31^^,^^118^ or old people^22^^,^^107^^,^^114^^,^^118^; gender (being female as more susceptible)^22^^,^^64^^,^^87^^,^^100^^,^^108^; having physical disabilities^118^ or pre-existing comorbidities (e.g., cardiovascular diseases, diabetes, obesity)^22^^,^^106^^,^^114^; and being a migrant.^118^

The geographical features of the natural or built environment^99^ were also identified as potential vulnerability factors, including living in steep slopes or mountain sides with the potential of landslides or erosion^96^; degraded, deforested, or deserted areas^96^^,^^104^^,^^111^; rural areas^97^^,^^100^; and lack of vegetation cover.^99^^,^^105^

More general social determinants of health^97^ also were identified as potential vulnerability factors. These cover: socioeconomic^95^^,^^100^^,^^103^^,^^106^^,^^108^^,^^113^^,^^116^ and sociodemographic^99^^,^^101^^,^^103^^,^^105^^,^^111^ determinants, including poverty,^101^^,^^102^^,^^105^ low income,^111^^,^^113^^,^^118^ population health status^103^^,^^104^ and distribution and population density,^98^^,^^112^ and human development level^100^^,^^107^; access to basic services, such as water^40^^,^^97^^,^^102^^,^^119^ or health services; social inequalities^97^^,^^106^; educational^64^ or literacy status^102^; and general living conditions.^102^^,^^119^

Finally, wider policy and institutional capacities were identified as factors that could potentially increase vulnerability, including, weak maintenance/management of basic services and infrastructure^95^^,^^108^; weak political engagement^118^; lack of public awareness^116^; lack of investment^116^; weak capacity to prepare, respond, adapt, and recover^101^^,^^103^^,^^115^^,^^116^^,^^119^; weak governance^109^^,^^110^; lack of planning^98^; poor risk communication.^110^^,^^117^

In terms of overall appraisal, most of the articles (n = 23) were suggested to be included^22^^,^^64^^,^^87^^,^^96^^,^^97^^,^^99^, ^100^, ^101^, ^102^, ^103^, ^104^, ^105^, ^106^, ^107^, ^108^, ^109^, ^110^^,^^113^^,^^115^, ^116^, ^117^, ^118^, ^119^; however, for seven articles^31^^,^^40^^,^^95^^,^^98^^,^^111^^,^^112^^,^^114^ the recommendation was to seek further information mainly because the vulnerability factor was not clearly stated, or it was not the main variable of analysis.

## Discussion

The assessment of the risk of negative outcomes due to climate hazards comprehend *i*) the presence and magnitude of climate hazards; *ii)* the level of population exposure to these climate hazards; and *iii)* population vulnerability which covers susceptibility and adaptive capacity. In this sense, it is desirable that adaptation and mitigation measures to protect health and wellbeing of populations consider a comprehensive evidence-based analysis of these three components of risk.

Evidence on these topics has been continuously growing in South America, allowing for a better comprehension of main climate hazards and impacts on population health. However, as it is demonstrated in this scoping review, there are still several gaps and research challenges on the intersection between climate change and population health and wellbeing, limiting further and deeper analyses of the health risks, especially analyses based on local data (see Panel 1).

Panel 1 **Gaps and research challenges.**

Although scientific evidence on these topics has increased over time in South America, several knowledge gaps and methodological issues still persist. While global information on the effects of climate change on population health is valuable, obtaining local data and knowledge is essential for developing effective adaptation policies. The impacts of climate change on health and wellbeing are mediated by local social vulnerabilities and adaptive capacities. Therefore, there is a critical need to gather local evidence on health impacts to adequately inform adaptation policies from a local perspective. Moreover, this scoping review and other complementary evidence indicate that a holistic perspective should be considered in understanding climate hazards, exposures, vulnerabilities, and health impacts, given the interwoven relationships between these elements. In order to comprehensively understand climate-sensitive health risks, a systemic approach should be taken to inform subsequent actions effectively.

These gaps and challenges do not only include the general lack of evidence and information, but also a lack of diversity in terms of disciplines and geographical coverage of research in the region. This situation might affect general knowledge on the topic and subsequent public awareness, as well as the decision-making processes related to mitigation and adaptation measures at different levels (national, regional, and local), and other climate-health political integration systems.

The evidence compiled in this study represents the differential research capacities in South American countries. Most of the evidence covers Argentina, Brazil, Chile, Colombia, and Peru, leaving countries with less research capacities behind. This is relevant as several hazards and ecological changes do not respect administrative boundaries and can create important public health challenges at the regional level. Therefore, strong international collaboration is needed to efficiently face climate change and population health challenges. Additionally, climate change is a complex issue that needs a systemic approach. Most of the evidence has been generated from a few disciplines, limiting the inclusion of other non-academic actors. Interdisciplinary and transdisciplinary research on climate and health is strongly needed in SA, allowing for building stronger links between academics, policymakers, policy implementers, and affected communities from different disciplines.^120^ This perspective would help addressing the gap in data generation and use, as well as translating scientific evidence into practice.

There are important gaps in terms of the methodologies. Ecological studies (those that analyse data at the population level) are an important and key tool to analyse climatic and health data; however, due to the intrinsic limitations of the ecological design, the evidence might not be useful to specific local areas where the climate hazards or population health status can be different. Second, comparability of studies in SA is very limited due to the use of different databases, or metrics, or methodologies, affecting the analysis of overall impacts, exposure degree, or vulnerability factors between and within countries. This latter issue might affect the decision-making processes at national level. A final challenge considers the use and availability of good quality databases and public health indicators. Information on these is scarce and varies between countries, affecting timely and reliable data analyses. This challenge might occur due to several reasons, including the weak integration of health institutions in each country; therefore, data are not timely integrated and quality-checked, or even it might be left incomplete temporally and spatially. It also might be happening because the digitalisation of health data is limited and has not been standardised between and within countries.

The strength of evidence presented multiple limitations. Most of the reviews were not systematic, and cited evidence of variable strength and quality, while most time series studies analysed annual or otherwise highly aggregated cases in a single or a few sites, limiting generalisability. Covariates did not include a comprehensive set of small-grid time-space climate-related factors nor it included vulnerability factors, and none assessed the intensity of disease control/prevention efforts. Reporting was also incomplete, often describing only significance and presence or absence of association without quantifying the strength of associations. Inclusion and exclusion criteria are rarely reported in sufficient detail, primarily using surveillance data “as is” without a proper understanding of its subtleties, critical in studying clinically diagnosed entities such as dengue, that often include a substantial fraction of non-dengue but “dengue-like” febrile illnesses. Also, limitations are scarcely described, as well as the implications of these limitations on the validity of the conclusions, preventing an accurate assessment of the quality of evidence. Earth sciences, public health concepts and methodology and statistical methods were not sufficiently integrated, and author’s affiliations do not reflect the extensive multidisciplinary or even transdisciplinary efforts needed to produce strong regional evidence.

Many scientific gaps exist, such as the impact of deglaciation on human health, studies on nutrition and food intake, understanding the mediating role of response interventions during emergencies and many others. Similarly, an important emphasis has been placed in the impacts of climate-related factors and El Niño–Southern Oscillation, but the consequences of highly relevant human-induced hazards in SA such as deforestation, floods, droughts, fires, and air pollution remain yet a significant gap in scientific knowledge. Also, long-term or even decade-long projections do not match political cycles and decision makers may appreciate shorter time-space scales. Also, while significance of most findings is high and correlations are strong, the standard error of results is also substantial and long-term projections have wide intervals, requiring careful interpretation for decision-making processes.

There are clear impacts of climate and its extremes on human health, including morbidity and mortality; however, more refined and accurate estimates are lacking. Also, the actual attributable impact of climate change has not been sufficiently assessed, leading to the question “*Do we know enough of the mechanisms and how they interact in the specific micro-scale of ecologically-differentiated regions?”* Maybe we are making too many assumptions based on ecological (highly aggregated) studies. More cohort studies such as Mal-Ed^41^ can better inform us of the mechanisms of climate-related events impact on health.

When analysing mental health impacts, this scoping review only obtained a small number of studies that complied with the stated eligibility criteria to respond to our research aim and objectives. Papers mainly reported data from Brazil, Peru, and Chile, or were non-systematic literature reviews that lacked clear reporting of outcomes. There were no intervention studies from our search. Two papers from Peru reported intra-familial violence amongst flood survivors and only one mentioned alcohol use in drought-affected groups. The diagnostic criteria and tools used to determine and assess the mental conditions under research were not consistent across the obtained studies. Reporting and publication bias seems likely as none reported negative mental health or wellbeing outcomes. Sample sizes also varied widely, from small qualitative studies to multi-country assessments. This study has identified disparate and minimal evidence based on climate change effects on mental health across South American countries, where underserved survivors of extreme events seem to be particularly disadvantaged. These different exposures to post-disaster stressors, in addition to the different support available across countries and the unique cultural and contextual factors, may interact in complex models, crucially impacting the individuals’ and group mental health responses and conditions. There is a concerning lack of formal assessments addressing these impacts with cultural and gender sensitivity, and community-based in mind. Furthermore, the methods and results of many included studies were frequently poorly reported, so methodological biases cannot be ruled out. This evidence paucity should be a call for action to address mental health and local factors with a transdisciplinary lens at all levels to translate them into policy and community engagement.

In terms of the analysis of exposure to climate hazards, evidence is scarce and limited in understanding the concept of population exposure that is generally mixed with vulnerability factors. This limited evidence might affect the study and identification of people highly exposed to hazards, which in turn limits the adaptation measures to reduce vulnerability. Therefore, it is important to spatially and temporally analyse to what extent population is exposed to hazards, identifying areas prone to be affected by the hazard as well as areas prone to disasters, where the link with social vulnerabilities is important.^121^ Additionally, most of the evidence analyses exposure to heat extremes and its consequences; however, the exposure to cold extremes is less studied and understood, leaving an important gap in terms of temperature exposure and the associated changes.

Regarding the identification of vulnerability or susceptibility factors, it is important to highlight that the scientific evidence identifies different factors at different levels, which is key to the correct identification of most vulnerable population and subsequent targeted actions. Individual susceptibility factors, such as age and comorbidities, and wider social determinants of health, such as socioeconomic status, have been identified in South American populations. Unfortunately, as most of these articles have taken an ecological approach, it might be difficult to assign specific risks to different populations. Public health practitioners or epidemiologists can take this information as an overall perspective and then analyse their own situations at local levels, but that would require specialised technical skills. Additionally, geographical and wider policy and political determinants are also identified, which are undoubtedly associated with the vulnerability of populations to climate hazards. In this case, these determinants are more associated with the capacities of institutions to respond, adapt, and recover from shocks or stresses, as well as the financial support and political will to progress in adaptation and mitigation measures. Unfortunately, evidence in SA has not integrated a general or standardised framework to understand and analyse vulnerability to climate hazards, leading to a wide range of definitions and approaches to susceptibility and adaptive capacities. There is a need for establishing a clear and useful framework that could guide the identification of vulnerable populations and subsequent policy measures. It is desirable that this framework would include a dynamic social approach to vulnerability given the multidimensional nature of population vulnerability to climate change.^122^

Based on the current published evidence, this is one of the first studies to apply a systematic approach to revise the scientific evidence on the three components of health risks associated with climate hazards. It marks a great precedent in the field of climate change and health and helps guides research to strengthen the practice and research on the field. Nonetheless, this study has some limitations. First, it did not include searches using Spanish and Portuguese key terms, which may have limited the number of articles and introduced some language biases. Second, the search was restricted to impacts, exposures, and vulnerabilities to climate change, excluding evidence related to impacts of climate, weather, or environmental hazards. This may have excluded several articles that only consider other environmental perspectives or frameworks. Additionally, as the search was restricted to climate and health intersection, other areas involving health-determining sectors may have been excluded as well.

In order to continue working on this area, the Working Group on Health Hazards, Exposures, and Impacts of the LCSA aims to track the health hazards, impacts, and exposures to climate hazards by quantifying and analysing sound and scientific-based indicators considering a regional perspective.

## Contributors

YKPS, AGL, ECF, YAE, SMH: conceptualization. YKPS, AGL, ECF, YAE, LR, MGC, WMR: investigation. YKPS, AGL, ECF, YAE, LR, MGC, WMR: formal analysis and data curation. YKPS, AGL, ECF, YAE: writing-original draft. YKPS, AGL, ECF, YAE, LR, MGC, WMR, SMH: writing-review.

## Declaration of interests

AGL is sponsored by Emerge, the Emerging Diseases Epidemiology Research Training grant D43 TW007393 awarded by the Fogarty International Center of the US National Institutes of Health. YKPS declares consultancy for the World Bank. All authors declare no conflicts of interest.

## Appendix A Supplementary data

Supplementary Tables S1–S4

Translated summary_ESP - disclaimer ok

Translated summary_POR - disclaimer ok
