# Supplementary material for: Identifying gaps on health impacts, exposures, and vulnerabilities to climate change on human health and wellbeing in South America: a scoping review
Source: Lancet Reg Health Am. 2023 Aug 24;26:100580. doi: 10.1016/j.lana.2023.100580 (PMC10593580; doi:10.1016/j.lana.2023.100580)
Supplement: Supplementary Tables S1–S4 [file mmc1.docx]

**SUPPLEMENTARY MATERIAL**

**Identifying Gaps on Health Impacts, Exposures, and Vulnerabilities to Climate Change on Human Health and Wellbeing in South America: A Scoping Review**

**Table S1.** Search strategies for: *What are the main impacts of climate change hazards on physical health and wellbeing in South America?*

| **Database: Web of Science** | | | |
| --- | --- | --- | --- |
| **Date: 14 November 2021** | | | |
| **Search** | **Strategy** | **Filters (if any)** | **Results** |
| 1 | TS=(“Climat* change$” OR “Climat* disrupt*” OR “Climat* hazard$” OR “Climat* extreme*” OR “Climat* catastroph*” OR “Climat* shift*” OR “Global warming” OR “Climat* variation$” OR “Climat* variab*” OR “Chang* climat*” OR “Weather change*” OR “Meteorol* chang*”) | None | 382,445 |
| 2 | TS=(“Temperature$ chang*” OR “Temperature* extreme$” OR “Temperature* anomal*” OR “Rising temperature*” OR “Temperature increase” OR “Temperature decrease” OR heat-wave OR Warm-spell OR Cold-spell OR “Extreme heat” OR “Extreme cold” OR “High temperature$” OR “Low temperature$”) | None | 841,421 |
| 3 | TS=(“Precipitation$” OR Moist OR Muggy OR Drought$ OR “Water scarcity” OR “Water stress” OR “Aridity” OR Dry-spell OR “Water Shortage$” OR “Palmer Drought Severity Index” OR Flood$ OR Inundation$ OR Deluge OR Downpour* OR Rain* OR Alluvion$) | None | 1,209,999 |
| 4 | TS=(“Extreme weather event$” OR “Extreme climat* event$” OR “Extreme hydrometeorological event$” OR “Extreme meteorological event$” OR Hurricane$ OR Tornado$ OR Typhoon$ OR Storm$ OR Windstorm$ OR “Tropical cyclone$”) | None | 139,900 |
| 5 | TS=(“Ocean acidification” OR “Sea acidification” OR “Ocean warming” OR “Ocean heat*” OR “El Ni$o” OR “Sea surface temperature” OR ENSO OR “ Southern Oscillation” OR “Sea level ris*” OR “Rising sea level*” OR “Coastal erosion” OR “Algae bloom*” OR “Brown tide” OR “Red tide” OR “Marine bloom” OR “Water bloom” OR “Black water” OR “Retreat of glacier*” OR “Melting sea ice” OR “Glacial meltwater*” OR “Glacier recession” OR “Reduced sea ice” OR “Ice sheet melt*” OR “Glacier shrink*” ) | None | 95,738 |
| 6 | TS=(biodiversity OR “Biodiversity change*” OR “Biodiversity loss*” OR “Specie* extinction” OR “Specie* loss” OR “Specie* disappearance” OR “Biological diversity loss*” OR “Ecosystem change*” OR “Vector* suitability” OR “Vector* adequacy” OR “Vector* capacity”) | None | 167,135 |
| 7 | TS=(Wild-fire$ OR Bush-fire$ OR “Forest fire$” OR “Wild-land fire*”) | None | 12,716 |
| 8 | TS=(health OR well-being OR quality-of-life OR welfare) | None | 3,156,161 |
| 9 | TS=(Death$ OR Mortal* OR Fatalit* OR Casualt* OR Disease$ OR Disorder$ OR Illness* OR Sickness* OR Indisposition$ OR Unhealth* OR Injur*) | None | 8,235,107 |
| 10 | TS=(Heat OR Heat-stroke$ OR Faintness OR “Warm skin” OR Dehydration OR Heat-stress OR “Heat exhaustion” OR “Heat hyperpyrexia” OR “Heat-related death$” OR “Respiratory disease$” OR “Vector-borne disease$” OR “Mosquito-borne Disease$” OR “Rodent-borne disease$” OR “Food-borne disease$” OR “Water-borne disease$” OR Poisoning) | None | 1,714,038 |
| 11 | TS=(“Food secur*” OR “Food scarcity” OR “Food shortage” OR “Food deprivation” OR “Food deficit” OR “Food shortfall” OR Malnutrit* OR Undernutri* OR Undernourish* OR Malnourish* OR Starv* OR Underfed OR Famine OR Hunger) | None | 184,790 |
| 12 | TS=(South-America* OR Argentin* OR Bolivia* OR Brazil* OR Chile* OR Colombia* OR Ecuador* OR Paraguay* OR Peru* OR Uruguay* OR Venezuela* OR Andes OR Amazon OR Altiplano OR Surinam* OR Guyan*) | None | 702,052 |
| 13 | TS=(human) | None | - |
| 14 | (#1 OR #2 OR #3 OR #4 OR #5 OR #6 OR #7) AND (#8 OR #9 OR #10 OR #11) AND #12 | None | - |
| 15 | #14 AND #13 | None | 395 |
| **Database: PubMed** | | | |
| **Date: 14 November 2021** | | | |
| **Search** | **Strategy** | **Filters (if any)** | **Results** |
| 1 | “Climate change”[TIAB] OR “Climate changes”[TIAB] OR “Climatic change”[TIAB] OR “Climatic changes”[TIAB] OR “Climate disruption”[TIAB] OR “Climate disruptions”[TIAB] OR “Climatic disruption”[TIAB] OR “Climatic disruptions”[TIAB] OR “Climate hazard”[TIAB] OR “Climate hazards”[TIAB] OR “Climatic hazard”[TIAB] OR “Climatic hazards”[TIAB] OR “Climate extreme”[TIAB] OR “Climate extremes”[TIAB] OR “Climatic extreme”[TIAB] OR “Climatic extremes”[TIAB] OR “Climate catastrophe”[TIAB] OR “Climate catastrophes”[TIAB] OR “Climatic catastrophe”[TIAB] OR “Climatic catastrophes”[TIAB] OR “Climate shift”[TIAB] OR “Climate shifts”[TIAB] OR “Climatic shift”[TIAB] OR “Climatic shifts”[TIAB] OR “Global warming”[TIAB] OR “Climate variation”[TIAB] OR “Climate variations”[TIAB] OR “Climatic variation”[TIAB] OR “Climatic variations”[TIAB] OR “Climate variability”[TIAB] OR “Climate variabilities”[TIAB] OR “Climatic variability”[TIAB] OR “Climatic variabilities”[TIAB] OR “Changing Climate”[TIAB] OR “Weather change”[TIAB] OR “Weather changes”[TIAB] OR “Meteorological change”[TIAB] |  | 57,543 |
| 2 | “Temperature changes”[TIAB] OR “Temperature change”[TIAB] OR “Temperature changing”[TIAB] OR “Temperature extreme”[TIAB] OR “Temperature extremes”[TIAB] OR “Temperature anomaly”[TIAB] OR “Temperature anomalies”[TIAB] OR “Rising temperature”[TIAB] OR “Rising temperatures”[TIAB] OR “Temperature increase”[TIAB] OR “Temperature increasing”[TIAB] OR “Temperature decrease”[TIAB] OR “Temperature decreasing”[TIAB] OR heat-wave[TIAB] OR heatwave[TIAB] OR “heat wave”[TIAB] OR warmspell[TIAB] OR warm-spell[TIAB] OR “Warm spell”[TIAB] OR “Warm spells”[TIAB] OR coldspell[TIAB] OR cold-spell[TIAB] OR “Cold spell”[TIAB] OR “Cold spells”[TIAB] OR “Extreme heat”[TIAB] OR “Extreme cold”[TIAB] OR “High temperature”[TIAB] OR “High temperatures”[TIAB] OR “Higher temperatures”[TIAB] OR “Higher temperature”[TIAB] OR “Low temperature”[TIAB] OR “Low temperatures”[TIAB] OR “Lower temperatures”[TIAB] OR “Lower temperature”[TIAB] |  | 143,297 |
| 3 | Precipitati*[TIAB] OR Moist*[TIAB] OR Evaporation[TIAB] OR Muggy[TIAB] OR Drought*[TIAB] OR “Water scarcity”[TIAB] OR “Water stress”[TIAB] OR “Aridity”[TIAB] OR dry-spell[TIAB] OR “Dry spell”[TIAB] OR “Water Shortage”[TIAB] OR “Palmer Drought Severity Index”[TIAB] OR Flood*[TIAB] OR Inundation*[TIAB] OR Deluge[TIAB] OR Downpour*[TIAB] OR Rain*[TIAB] OR Alluvion*[TIAB] |  | 247,308 |
| 4 | “Extreme weather event”[TIAB] OR “Extreme weather events”[TIAB] OR “Extreme climate event”[TIAB] OR “Extreme climatic event”[TIAB] OR “Extreme climate events”[TIAB] OR “Extreme climate event”[TIAB] OR “Extreme hydrometeorological event”[TIAB] OR “Extreme meteorological events”[TIAB] OR Hurricane*[TIAB] OR Tornado*[TIAB] OR Typhoon*[TIAB] OR Storm*[TIAB] OR Windstorm*[TIAB] OR “Tropical cyclone”[TIAB] OR “Tropical cyclones”[TIAB] |  | 25,122 |
| 5 | “Ocean acidification”[TIAB] OR “Ocean warming”[TIAB] OR “Ocean heat”[TIAB] OR “Ocean heating”[TIAB] OR “El Nino”[TIAB] OR “El Niño”[TIAB] OR “Sea surface temperature”[TIAB] OR ENSO[TIAB] OR “Southern Oscillation”[TIAB] OR “Sea level rise”[TIAB] OR “Sea level rising”[TIAB] OR “Rising sea level”[TIAB] OR “Rising sea levels”[TIAB] OR “Coastal erosion”[TIAB] OR “Algae bloom”[TIAB] OR “Brown tide”[TIAB] OR “Red tide”[TIAB] OR “Marine bloom”[TIAB] OR “Water bloom”[TIAB] OR “Black water”[TIAB] OR “Retreat of glacier”[TIAB] OR “Retreat of glaciers”[TIAB] OR “Melting sea ice”[TIAB] OR “Glacial meltwater”[TIAB] OR “Glacier recession”[TIAB] OR “Reduced sea ice”[TIAB] OR “Ice sheet melt”[TIAB] OR “Ice sheet melting”[TIAB] OR “Glacier shrinking”[TIAB] OR “Glacier shrinkage”[TIAB] |  | 9,203 |
| 6 | biodiversity[TIAB] OR “Biodiversity change”[TIAB] OR “Biodiversity changes”[TIAB] OR “Biodiversity loss”[TIAB] OR “Specie extinction”[TIAB] OR “Species extinction”[TIAB] OR “Specie extinctions”[TIAB] OR “Species extinctions”[TIAB] OR “Specie loss”[TIAB] OR “Species loss”[TIAB] OR “Specie disappearance”[TIAB] OR “Species disappearance”[TIAB] OR “Biological diversity loss” [TIAB] OR “Ecosystem change”[TIAB] OR “Ecosystem changes”[TIAB] OR “Vector suitability”[TIAB] OR “Vector adequacy”[TIAB] OR “Vector capacity”[TIAB] OR “vectorial suitability”[TIAB] OR “vectorial adequacy”[TIAB] OR “vectorial capacity”[TIAB] |  | 35,054 |
| 7 | “Wild fire”[TIAB] OR “Wild fires”[TIAB] OR wild-fire*[TIAB] OR Wildfire*[TIAB] OR “Bush fire”[TIAB] OR “Bush fires”[TIAB] OR bushfire*[TIAB] OR bush-fire*[TIAB] OR “Forest fire”[TIAB] OR “Forest fires”[TIAB] OR “Wild-land fire”[TIAB] OR “Wild-land fires”[TIAB] |  | 3,956 |
| 8 | health[TIAB] OR well-being[TIAB] OR wellbeing[TIAB] OR  quality-of-life[TIAB] OR “quality of life”[TIAB] OR welfare[TIAB] |  | 2,459,381 |
| 9 | Death*[TIAB] OR Mortal*[TIAB] OR Fatalit*[TIAB] OR Casualt*[TIAB] OR Disease*[TIAB] OR Disorder*[TIAB] OR Illness*[TIAB] OR Sick*[TIAB] OR Indisposition*[TIAB] OR Unhealth*[TIAB] OR Injur*[TIAB] |  | 7,134,879 |
| 10 | Heat*[TIAB] OR heat-stroke*[TIAB] OR “Heat stroke”[TIAB] OR “Heat strokes”[TIAB] OR Faintness[TIAB] OR “Warm skin”[TIAB] OR Dehydration[TIAB] OR heat-stress[TIAB] OR “Heat stress”[TIAB] OR “Heat exhaustion”[TIAB] OR “Heat hyperpyrexia”[TIAB] OR “Heat-related death”[TIAB] OR “Heat related death”[TIAB] OR “Heat-related deaths”[TIAB] OR “Heat related deaths”[TIAB] OR “Respiratory disease”[TIAB] OR “Respiratory diseases”[TIAB] OR “Vector borne disease”[TIAB] OR “Vector-borne disease”[TIAB] OR “Vector borne diseases”[TIAB] OR “Vector-borne diseases”[TIAB] OR “Mosquito borne disease”[TIAB] OR “Mosquito-borne disease”[TIAB] OR “Mosquito borne diseases”[TIAB] OR “Mosquito-borne diseases”[TIAB] OR “Rodent borne disease”[TIAB] OR “Rodent-borne disease”[TIAB] OR “Rodent borne diseases”[TIAB] OR “Rodent-borne diseases”[TIAB] OR “Food borne disease”[TIAB] OR “Food-borne disease”[TIAB] OR “Food borne diseases”[TIAB] OR “Food-borne diseases”[TIAB] OR “Water borne disease”[TIAB] OR “Water-borne disease”[TIAB] OR “Water borne diseases”[TIAB] OR “Water-borne diseases”[TIAB] OR Poisoning[TIAB] |  | 450,543 |
| 11 | “Food security”[TIAB] OR “Food scarcity”[TIAB] OR “Food shortage”[TIAB] OR “Food deprivation”[TIAB] OR “Food deficit”[TIAB] OR “Food shortfall”[TIAB] OR Malnutrition*[TIAB] OR Undernutrition[TIAB] OR Undernourish*[TIAB] OR Malnourish*[TIAB] OR Starv*[TIAB] OR Underfed*[TIAB] OR Famine[TIAB] OR Hunger[TIAB] |  | 121,380 |
| 12 | South-America*[TIAB] OR South America*[TIAB] OR Argentin*[TIAB] OR Bolivia*[TIAB] OR Brazil*[TIAB] OR Chile*[TIAB] OR Colombia*[TIAB] OR Ecuador*[TIAB] OR Paraguay*[TIAB] OR Peru*[TIAB] OR Uruguay*[TIAB] OR Venezuela*[TIAB] OR Ande*[TIAB] OR Amazon[TIAB] OR Altiplano[TIAB] OR Surinam*[TIAB] OR Guyan*[TIAB] |  | 251,703 |
| 13 | #2 OR #3 OR #4 OR #5 OR #6 OR #7 |  | 443,940 |
| 14 | #8 OR #9 OR #10 OR #11 |  | 9,043,605 |
| 15 | #1 AND #13 AND #14 AND #12 | Humans | 96 |
| **Database: PubMed MeSH** | | | |
| **Date: 14 November 2021** | | | |
| **Search** | **Strategy** | **Filters (if any)** | **Results** |
| 1 | ("Global Warming"[Mesh]) OR "Climate Change"[Mesh] |  | 23,906 |
| 2 | ((((("Extreme Heat"[Mesh]) OR "Cold Temperature"[Mesh]) OR "Hot Temperature"[Mesh]) OR "Extreme Hot Weather"[Mesh]) OR "Extreme Cold Weather"[Mesh]) OR "Extreme Weather"[Mesh] |  | 190,930 |
| 3 | (((("Rain"[Mesh]) OR "Humidity"[Mesh]) OR "Droughts"[Mesh]) OR "Floods"[Mesh]) OR "Water Insecurity"[Mesh] |  | 40,864 |
| 4 | (("Extreme Weather"[Mesh]) OR "Cyclonic Storms"[Mesh]) OR "Tornadoes"[Mesh] |  | 2,982 |
| 5 | (("El Nino-Southern Oscillation"[Mesh]) OR "Sea Level Rise"[Mesh]) OR "Harmful Algal Bloom"[Mesh] |  | 1,880 |
| 6 | ("Biodiversity"[Mesh]) OR "Extinction, Biological"[Mesh] |  | 104,059 |
| 7 | "Wildfires"[Mesh] |  | 702 |
| 8 | ("Health"[Mesh]) OR "Quality of Life"[Mesh] |  | 612,439 |
| 9 | (("Death"[Mesh]) OR "Mortality"[Mesh]) OR "Wounds and Injuries"[Mesh] |  | 1,464,704 |
| 10 | ((((((("Heat Stroke"[Mesh]) OR "Dehydration"[Mesh]) OR "Heat Stress Disorders"[Mesh]) OR "Heat Exhaustion"[Mesh]) OR "Respiratory Tract Diseases"[Mesh]) OR "Vector Borne Diseases"[Mesh]) OR "Foodborne Diseases"[Mesh]) OR "Poisoning"[Mesh] (((("Food Security"[Mesh]) OR "Food Deprivation"[Mesh]) OR "Malnutrition"[Mesh]) OR "Famine"[Mesh]) OR "Hunger"[Mesh] |  | 22,523 |
| 11 | (((((((((((("South America"[Mesh]) OR "Argentina"[Mesh]) OR "Bolivia"[Mesh]) OR "Brazil"[Mesh]) OR "Chile"[Mesh]) OR "Colombia"[Mesh]) OR "Ecuador"[Mesh]) OR "Paraguay"[Mesh]) OR "Peru"[Mesh]) OR "Suriname"[Mesh]) OR "Uruguay"[Mesh]) OR "Venezuela"[Mesh]) OR "Guyana"[Mesh] |  | 180,741 |
| 12 | #2 OR #3 OR #4 OR #5 OR #6 OR #7 |  | 335,061 |
| 13 | #8 OR #9 OR #10 |  | 2,061,716 |
| 14 | #1 AND #12 AND #13 AND #11 | Humans | 11 |
| **Database: ProQuest** | | | |
| **Date: 14 November 2021** | | | |
| **Search** | **Strategy** | **Filters (if any)** | **Results** |
| 1 | ab("Climat* change$" OR "Climat* disrupt*" OR "Climat* hazard$" OR "Climat* extreme*" OR "Climat* catastroph*" OR "Climat* shift*" OR "Global warming" OR "Climat* variation$" OR "Climat* variab*" OR "Chang* climat*" OR "Weather change*" OR "Meteorol* chang*") AND ab("Temperature$ chang*" OR "Temperature* extreme$" OR "Temperature* anomal*" OR "Rising temperature*" OR "Temperature increase" OR "Temperature decrease" OR heat-wave OR Warm-spell OR Cold-spell OR "Extreme heat" OR "Extreme cold" OR "High temperature$" OR "Low temperature$" OR "Precipitation$" OR Moist OR Muggy OR Drought OR "Water scarcity" OR "Water stress" OR "Aridity" OR Dry-spell OR "Water Shortage$" OR "Palmer Drought Severity Index" OR Flood OR Inundation OR Deluge OR Downpour* OR Rain* OR Alluvion OR "Extreme weather event$" OR "Extreme climat* event$" OR "Extreme hydrometeorological event$" OR "Extreme meteorological event$" OR Hurricane OR Tornado OR Typhoon OR Storm OR Windstorm OR "Tropical cyclone$" OR "Ocean acidification" OR "Sea acidification" OR "Ocean warming" OR "Ocean heat*" OR "Sea heat*" OR "Sea warming" OR "El Ni$o" OR "Sea surface temperature" OR ENSO OR " Southern Oscillation" OR "Sea level ris*" OR "Rising sea level*" OR "Coastal erosion" OR "Algae bloom*" OR "Brown tide" OR "Red tide" OR "Marine bloom" OR "Water bloom" OR "Black water" OR "Retreat of glacier*" OR "Melting sea ice" OR "Glacial meltwater*" OR "Glacier recession" OR "Reduced sea ice" OR "Ice sheet melt*" OR "Glacier shrink*" OR biodiversity OR "Biodiversity change*" OR "Biodiversity loss*" OR "Specie* extinction" OR "Specie* loss" OR "Specie* disappearance" OR "Biological diversity loss*" OR "Ecosystem change*" OR "Vector* suitability" OR "Vector* adequacy" OR "Vector* capacity" OR Wild-fire OR Bush-fire OR "Forest fire$" OR "Wild-land fire*") AND ab(health OR well-being OR quality-of-life OR welfare OR Death OR Mortal* OR Fatalit* OR Casualt* OR Disease OR Disorder OR Illness* OR Sickness* OR Indisposition OR Unhealth* OR Injur* OR Heat OR Heat-stroke OR Faintness OR "Warm skin" OR Dehydration OR Heat-stress OR "Heat exhaustion" OR "Heat hyperpyrexia" OR "Heat-related death$" OR "Respiratory disease$" OR "Vector-borne disease$" OR "Mosquito-borne Disease$" OR "Rodent-borne disease$" OR "Food-borne disease$" OR "Water-borne disease$" OR Poisoning OR "Food secur*" OR "Food scarcity" OR "Food shortage" OR "Food deprivation" OR "Food deficit" OR "Food shortfall" OR Malnutrit* OR Undernutri* OR Undernourish* OR Malnourish* OR Starv* OR Underfed OR Famine OR Hunger) AND ab(South-America* OR Argentin* OR Bolivia* OR Brazil* OR Chile* OR Colombia* OR Ecuador* OR Paraguay* OR Peru* OR Uruguay* OR Venezuela* OR Andes OR Amazon OR Altiplano OR Surinam* OR Guyan*) AND ab(human) | Peer review | 97 |
| **Database: Scopus** | | | |
| **Date: 14 November 2021** | | | |
| **Search** | **Strategy** | **Filters (if any)** | **Results** |
| 1 | ( ABS ( {Climate change} OR {Climate disruption} OR {Climate hazard} OR {Climate extreme} OR {Climate catastrophe} OR {Climate shift} OR {Global warming} OR {Climate variation} OR {Climate variability} OR {Changing climate} OR {Weather change} OR {Meteorological change} ) AND ABS ( {Temperature change} OR {Temperature extreme} OR {Temperature anomaly} OR {Rising temperature} OR {Temperature increase} OR {Temperature decrease} OR heat-wave OR warm-spell OR cold-spell OR {Extreme heat} OR {Extreme cold} OR {High temperature} OR {Low temperature} OR precipitation* OR {Humidity change} OR mold OR moist OR evaporation OR muggy OR drought* OR {Water scarcity} OR {Water stress} OR aridity OR dry-spell OR {Water Shortage} OR {Palmer Drought Severity Index} OR flood* OR inundation* OR deluge OR downpour* OR rain* OR alluvion* OR {Extreme weather event} OR {Extreme climate event} OR {Extreme hydrometeorological event} OR {Extreme meteorological event} OR hurricane* OR tornado* OR typhoon* OR storm* OR windstorm* OR {Tropical cyclone} OR {ocean acidification} OR {sea acidification} OR {ocean warming} OR {ocean heating} OR {sea heating} OR {sea warming} OR {el nino} OR {el niño} OR {sea surface temperature} OR enso OR {southern oscillation} OR {sea level rise} OR {rising sea level} OR {coastal erosion} OR {algae bloom} OR {brown tide} OR {red tide} OR {marine bloom} OR {water bloom} OR {black water} OR {retreat of glacier} OR {melting sea ice} OR {glacial meltwater} OR {glacier recession} OR {reduced sea ice} OR {ice sheet melting} OR {glacier shrink} OR biodiversity OR {Biodiversity change} OR {Biodiversity loss} OR {Specie extinction} OR {Specie loss} OR {Specie disappearance} OR {Biological diversity loss} OR {Ecosystem change} OR {Vector suitability} OR {Vector adequacy} OR {Vector capacity} OR wild-fire OR wildfire OR bush-fire OR bushfire OR {Forest fire} OR {Wild-land fire} ) AND ABS ( health OR well-being OR {quality-of-life} OR {quality of life} OR welfare OR death OR mortal* OR fatalit* OR casualt* OR disease* OR disorder* OR illness* OR sickness* OR indisposition* OR unhealth* OR injur* OR heat OR heat-stroke OR faintness OR {Warm skin} OR dehydration OR heat-stress OR {Heat exhaustion} OR {Heat hyperpyrexia} OR {Heat-related death} OR {health-related mortality} OR {health-related disease} OR {Respiratory disease} OR {Vector-borne disease} OR {Mosquito-borne Disease} OR {Rodent-borne disease} OR {Food-borne disease} OR {Water-borne disease} OR poisoning OR {Food security} OR {Food scarcity} OR {Food shortage} OR {Food deprivation} OR {Food deficit} OR {Food shortfall} OR malnutrit* OR undernutri* OR undernourish* OR malnourish* OR starv* OR underfed OR famine OR hunger ) AND ABS ( {South-America*} OR argentin* OR bolivia* OR brazil* OR chile* OR colombia* OR ecuador* OR paraguay* OR peru* OR uruguay* OR venezuela* OR andes OR amazon OR altiplano OR surinam* OR guyan* ) AND ABS ( {human health} ) ) |  | 25 |
| **Database: SciELO** | | | |
| **Date: 14 November 2021** | | | |
| **Search** | **Strategy** | **Filters (if any)** | **Results** |
|  | (("climate change" OR "climate disruption" OR "climate hazard" OR "climate extreme" OR "climate catastrophe" OR "climate shift" OR "global warming" OR "climate variation" OR "climate variability" OR "changing climate" OR "weather change" OR "meteorological change") OR ("temperature change" OR "temperature extreme" OR "temperature anomaly" OR "rising temperature" OR "temperature increase" OR "temperature decrease" OR heat-wave OR warm-spell OR cold-spell OR "extreme heat" OR "extreme cold" OR "high temperature" OR "low temperature") OR (precipitation OR "humidity change" OR mold OR moist OR evaporation OR muggy OR drought OR "water scarcity" OR "water stress" OR aridity OR dry-spell OR "water shortage" OR "palmer drought severity index" OR flood OR inundation OR deluge OR downpour OR rain OR alluvion) OR ("extreme weather event" OR "extreme climate event" OR "extreme meteorological event" OR "extreme hydrometeorological event" OR hurricane OR tornado OR typhoon OR storm OR windstorm OR "tropical cyclone") OR ("ocean acidification" OR "sea acidification" OR "ocean warming" OR "ocean heating" OR "sea heating" OR "sea warming" OR "el nino" OR "el niño" OR "sea surface temperature" OR enso OR "southern oscillation" OR "sea level rise" OR "rising sea level" OR "coastal erosion" OR "algae bloom" OR "brown tide" OR "red tide" OR "marine bloom" OR "water bloom" OR "black water" OR "retreat of glacier" OR "melting sea ice" OR "glacial meltwater" OR "glacier recession" OR "reduced sea ice" OR "ice sheet melting" OR "glacier shrink") OR (biodiversity OR "biodiversity change" OR "biodiversity loss" OR "specie extintion" OR "specie loss" OR "specie disappearance" OR "biological diversity loss" OR "ecosystem change" OR "vector suitability" OR "vector adequacy" OR "vector capacity") OR (wild-fire OR wildfire OR bush-fire OR bushfire OR "forest fire" OR "wild land fire")) AND ((health OR well-being OR "quality-of-life" OR "quality of life" OR welfare OR death OR mortalit* OR fatalit* OR casualt* OR disease* OR disorder* OR illness* OR sickness* OR indisposition* OR unhealth* OR injur*) OR (heat OR heat-stroke OR faintness OR "warm skin" OR dehydration OR heat-stress OR "heat exhaustion" OR "heat hyperpyrexia" OR "heat-related death" OR "heat-related mortality" OR "health-related disease" OR "respiratory disease" OR "vector-borne disease" OR "mosquito-borne disease" OR "rodent-borne disease" OR "food-borne disease" OR "water-borne disease" OR poisoning OR "food security" OR "food scarcity" OR "food shortage" OR "food deprivation" OR "food deficit" OR "food shortfall" OR malnutrit* OR undernutrit* OR undernourish* OR malnourish* OR starv* OR underfed* OR famine OR hunger)) AND ("South America" OR South-America* OR argentin* OR bolivia* OR brazil* OR chile* OR colombia* OR ecuador* OR paraguay* OR peru* OR uruguay* OR venezuela* OR andes OR amazon OR altiplano OR surinam* OR guyan*) AND (human) |  | 72 |
| **Database: BIREME/LILACS** | | | |
| **Date: 14 November 2021** | | | |
| **Search** | **Strategy** | **Filters (if any)** | **Results** |
| 1 | (ab:(("climate change") OR ("climate disruption") OR ("climate hazard") OR ("climate extreme") OR ("climate catastrophe") OR ("climate shift") OR ("global warming") OR ("climate variation") OR ("climate variability") OR ("changing climate") OR ("weather change") OR ("meteorological change") OR ("temperature change") OR ("temperature extreme") OR ("temperature anomaly") OR ("rising temperature") OR ("temperature increase") OR ("temperature decrease") OR (heat-wave) OR (warm-spell) OR (cold-spell) OR ("extreme heat") OR ("extreme cold") OR ("high temperature") OR ("low temperature") OR (precipitation) OR ("humidity change") OR (mold) OR (moist) OR (evaporation) OR (muggy) OR (drought) OR ("water scarcity") OR ("water stress") OR (aridity) OR (dry-spell) OR ("water shortage") OR ("palmer drought severity index") OR (flood) OR (inundation) OR (deluge) OR (downpour) OR (rain) OR (alluvion) OR ("extreme weather event") OR ("extreme climate event") OR ("extreme meteorological event") OR ("extreme hydrometeorological event") OR (hurricane) OR (tornado) OR (typhoon) OR (storm) OR (windstorm) OR ("tropical cyclone") OR ("ocean acidification") OR ("sea acidification") OR ("ocean warming") OR ("ocean heating") OR ("sea heating") OR ("sea warming") OR ("el nino") OR ("el niño") OR ("sea surface temperature") OR (enso) OR ("southern oscillation") OR ("sea level rise") OR ("rising sea level") OR ("coastal erosion") OR ("algae bloom") OR ("brown tide") OR ("red tide") OR ("marine bloom") OR ("water bloom") OR ("black water") OR ("retreat of glacier") OR ("melting sea ice") OR ("glacial meltwater") OR ("glacier recession") OR ("reduced sea ice") OR ("ice sheet melting") OR ("glacier shrink") OR (biodiversity) OR ("biodiversity change") OR ("biodiversity loss") OR ("specie extintion") OR ("specie loss") OR ("specie disappearance") OR ("biological diversity loss") OR ("ecosystem change") OR ("vector suitability") OR ("vector adequacy") OR ("vector capacity") OR (wild-fire) OR (wildfire) OR (bush-fire) OR (bushfire) OR ("forest fire") OR ("wild land fire"))) AND (ab:((health) OR (well-being) OR ("quality-of-life") OR ("quality of life") OR (welfare) OR (death) OR (mortality) OR (fatality) OR (casualty) OR (disease) OR (disorder) OR (illness) OR (sickness) OR (indisposition) OR (unhealth) OR (injury) OR (heat) OR (heat-stroke) OR (faintness) OR ("warm skin") OR (dehydration) OR (heat-stress) OR ("heat exhaustion") OR ("heat hyperpyrexia") OR ("heat-related death") OR ("heat-related mortality") OR ("health-related disease") OR ("respiratory disease") OR ("vector-borne disease") OR ("mosquito-borne disease") OR ("rodent-borne disease") OR ("food-borne disease") OR ("water-borne disease") OR (poisoning) OR ("food security") OR ("food scarcity") OR ("food shortage") OR ("food deprivation") OR ("food deficit") OR ("food shortfall") OR (malnutrition) OR (undernutrition) OR (undernourish) OR (malnourish) OR (starvation) OR (underfed) OR (famine) OR (hunger))) AND (ab:(("South America") OR (South-America) OR (argentina) OR (bolivia) OR (brazil) OR (chile) OR (colombia) OR (ecuador) OR (paraguay) OR (peru) OR (uruguay) OR (venezuela) OR (andes) OR (amazon) OR (altiplano) OR (suriname) OR (guyana))) AND (human) |  | 17 |

**Table S2.** Search strategies for: *What are the main impacts of climate change hazards on mental health and wellbeing in South America?*

| **Database: Web of Science** | | | |
| --- | --- | --- | --- |
| **Date: 31 October 2021** | | | |
| **Search** | **Strategy** | **Filters (if any)** | **Results** |
| 1 | TS=(“Climat* change$” OR “Climat* disrupt*” OR “Climat* hazard$” OR “Climat* extreme*” OR “Climat* catastroph*” OR “Climat* shift*” OR “Global warming” OR “Climat* variation$” OR “Climat* variab*” OR “Chang* climat*” OR “Weather change*” OR “Meteorol* chang*”) | None | 382,445 |
| 2 | TS=(“Temperature$ chang*” OR “Temperature* extreme$” OR “Temperature* anomal*” OR “Rising temperature*” OR “Temperature increase” OR “Temperature decrease” OR heat-wave OR Warm-spell OR Cold-spell OR “Extreme heat” OR “Extreme cold” OR “High temperature$” OR “Low temperature$”) | None | 841,421 |
| 3 | TS=(“Precipitation$” OR Moist OR Muggy OR Drought$ OR “Water scarcity” OR “Water stress” OR “Aridity” OR Dry-spell OR “Water Shortage$” OR “Palmer Drought Severity Index” OR Flood$ OR Inundation$ OR Deluge OR Downpour* OR Rain* OR Alluvion$) | None | 1,209,999 |
| 4 | TS=(“Extreme weather event$” OR “Extreme climat* event$” OR “Extreme hydrometeorological event$” OR “Extreme meteorological event$” OR Hurricane$ OR Tornado$ OR Typhoon$ OR Storm$ OR Windstorm$ OR “Tropical cyclone$”) | None | 139,900 |
| 5 | TS=(“Ocean acidification” OR “Sea acidification” OR “Ocean warming” OR “Ocean heat*” OR “El Ni$o” OR “Sea surface temperature” OR ENSO OR “ Southern Oscillation” OR “Sea level ris*” OR “Rising sea level*” OR “Coastal erosion” OR “Algae bloom*” OR “Brown tide” OR “Red tide” OR “Marine bloom” OR “Water bloom” OR “Black water” OR “Retreat of glacier*” OR “Melting sea ice” OR “Glacial meltwater*” OR “Glacier recession” OR “Reduced sea ice” OR “Ice sheet melt*” OR “Glacier shrink*” ) | None | 95,738 |
| 6 | TS=(biodiversity OR “Biodiversity change*” OR “Biodiversity loss*” OR “Specie* extinction” OR “Specie* loss” OR “Specie* disappearance” OR “Biological diversity loss*” OR “Ecosystem change*” OR “Vector* suitability” OR “Vector* adequacy” OR “Vector* capacity”) | None | 167,135 |
| 7 | TS=(Wild-fire$ OR Bush-fire$ OR “Forest fire$” OR “Wild-land fire*”) | None | 12,716 |
| 8 | TS=(“Mental health” OR “Mental disorder” OR “Post-traumatic stress disorder” OR Anxiet* OR Depress* OR Solastalgia OR Eco?anxi* OR Ecoguilt* OR “Ecological grief” OR “Biospheric concern”) | None | 692,662 |
| 9 | TS=(Displacement$ OR Migration$ OR Migrant$) | None |  |
| 10 | TS=(South-America* OR Argentin* OR Bolivia* OR Brazil* OR Chile* OR Colombia* OR Ecuador* OR Paraguay* OR Peru* OR Uruguay* OR Venezuela* OR Andes OR Amazon OR Altiplano OR Surinam* OR Guyan*) | None | 702,052 |
| 11 | TS=(human) | None | - |
| 12 | (#1 OR #2 OR #3 OR #4 OR #5 OR #6 OR #7) AND (#8 OR #9) AND #10 | None | 3,213 |
| 13 | #11 AND #12 |  | 331 |
| **Database: Pubmed** | | | |
| **Date: 24 October 2021** | | | |
| **Search** | **Strategy** | **Filters (if any)** | **Results** |
| 1 | “Climate change”[TIAB] OR “Climate changes”[TIAB] OR “Climatic change”[TIAB] OR “Climatic changes”[TIAB] OR “Climate disruption”[TIAB] OR “Climate disruptions”[TIAB] OR “Climatic disruption”[TIAB] OR “Climatic disruptions”[TIAB] OR “Climate hazard”[TIAB] OR “Climate hazards”[TIAB] OR “Climatic hazard”[TIAB] OR “Climatic hazards”[TIAB] OR “Climate extreme”[TIAB] OR “Climate extremes”[TIAB] OR “Climatic extreme”[TIAB] OR “Climatic extremes”[TIAB] OR “Climate catastrophe”[TIAB] OR “Climate catastrophes”[TIAB] OR “Climatic catastrophe”[TIAB] OR “Climatic catastrophes”[TIAB] OR “Climate shift”[TIAB] OR “Climate shifts”[TIAB] OR “Climatic shift”[TIAB] OR “Climatic shifts”[TIAB] OR “Global warming”[TIAB] OR “Climate variation”[TIAB] OR “Climate variations”[TIAB] OR “Climatic variation”[TIAB] OR “Climatic variations”[TIAB] OR “Climate variability”[TIAB] OR “Climate variabilities”[TIAB] OR “Climatic variability”[TIAB] OR “Climatic variabilities”[TIAB] OR “Changing Climate”[TIAB] OR “Weather change”[TIAB] OR “Weather changes”[TIAB] OR “Meteorological change”[TIAB] |  | 57,543 |
| 2 | “Temperature changes”[TIAB] OR “Temperature change”[TIAB] OR “Temperature changing”[TIAB] OR “Temperature extreme”[TIAB] OR “Temperature extremes”[TIAB] OR “Temperature anomaly”[TIAB] OR “Temperature anomalies”[TIAB] OR “Rising temperature”[TIAB] OR “Rising temperatures”[TIAB] OR “Temperature increase”[TIAB] OR “Temperature increasing”[TIAB] OR “Temperature decrease”[TIAB] OR “Temperature decreasing”[TIAB] OR heat-wave[TIAB] OR heatwave[TIAB] OR “heat wave”[TIAB] OR warmspell[TIAB] OR warm-spell[TIAB] OR “Warm spell”[TIAB] OR “Warm spells”[TIAB] OR coldspell[TIAB] OR cold-spell[TIAB] OR “Cold spell”[TIAB] OR “Cold spells”[TIAB] OR “Extreme heat”[TIAB] OR “Extreme cold”[TIAB] OR “High temperature”[TIAB] OR “High temperatures”[TIAB] OR “Higher temperatures”[TIAB] OR “Higher temperature”[TIAB] OR “Low temperature”[TIAB] OR “Low temperatures”[TIAB] OR “Lower temperatures”[TIAB] OR “Lower temperature”[TIAB] |  | 143,297 |
| 3 | Precipitati*[TIAB] OR Moist*[TIAB] OR Evaporation[TIAB] OR Muggy[TIAB] OR Drought*[TIAB] OR “Water scarcity”[TIAB] OR “Water stress”[TIAB] OR “Aridity”[TIAB] OR dry-spell[TIAB] OR “Dry spell”[TIAB] OR “Water Shortage”[TIAB] OR “Palmer Drought Severity Index”[TIAB] OR Flood*[TIAB] OR Inundation*[TIAB] OR Deluge[TIAB] OR Downpour*[TIAB] OR Rain*[TIAB] OR Alluvion*[TIAB] |  | 247,308 |
| 4 | “Extreme weather event”[TIAB] OR “Extreme weather events”[TIAB] OR “Extreme climate event”[TIAB] OR “Extreme climatic event”[TIAB] OR “Extreme climate events”[TIAB] OR “Extreme climate event”[TIAB] OR “Extreme hydrometeorological event”[TIAB] OR “Extreme meteorological events”[TIAB] OR Hurricane*[TIAB] OR Tornado*[TIAB] OR Typhoon*[TIAB] OR Storm*[TIAB] OR Windstorm*[TIAB] OR “Tropical cyclone”[TIAB] OR “Tropical cyclones”[TIAB] |  | 25,122 |
| 5 | “Ocean acidification”[TIAB] OR “Ocean warming”[TIAB] OR “Ocean heat”[TIAB] OR “Ocean heating”[TIAB] OR “El Nino”[TIAB] OR “El Niño”[TIAB] OR “Sea surface temperature”[TIAB] OR ENSO[TIAB] OR “Southern Oscillation”[TIAB] OR “Sea level rise”[TIAB] OR “Sea level rising”[TIAB] OR “Rising sea level”[TIAB] OR “Rising sea levels”[TIAB] OR “Coastal erosion”[TIAB] OR “Algae bloom”[TIAB] OR “Brown tide”[TIAB] OR “Red tide”[TIAB] OR “Marine bloom”[TIAB] OR “Water bloom”[TIAB] OR “Black water”[TIAB] OR “Retreat of glacier”[TIAB] OR “Retreat of glaciers”[TIAB] OR “Melting sea ice”[TIAB] OR “Glacial meltwater”[TIAB] OR “Glacier recession”[TIAB] OR “Reduced sea ice”[TIAB] OR “Ice sheet melt”[TIAB] OR “Ice sheet melting”[TIAB] OR “Glacier shrinking”[TIAB] OR “Glacier shrinkage”[TIAB] |  | 9,203 |
| 6 | biodiversity[TIAB] OR “Biodiversity change”[TIAB] OR “Biodiversity changes”[TIAB] OR “Biodiversity loss”[TIAB] OR “Specie extinction”[TIAB] OR “Species extinction”[TIAB] OR “Specie extinctions”[TIAB] OR “Species extinctions”[TIAB] OR “Specie loss”[TIAB] OR “Species loss”[TIAB] OR “Specie disappearance”[TIAB] OR “Species disappearance”[TIAB] OR “Biological diversity loss” [TIAB] OR “Ecosystem change”[TIAB] OR “Ecosystem changes”[TIAB] OR “Vector suitability”[TIAB] OR “Vector adequacy”[TIAB] OR “Vector capacity”[TIAB] OR “vectorial suitability”[TIAB] OR “vectorial adequacy”[TIAB] OR “vectorial capacity”[TIAB] |  | 35,054 |
| 7 | “Wild fire”[TIAB] OR “Wild fires”[TIAB] OR wild-fire*[TIAB] OR Wildfire*[TIAB] OR “Bush fire”[TIAB] OR “Bush fires”[TIAB] OR bushfire*[TIAB] OR bush-fire*[TIAB] OR “Forest fire”[TIAB] OR “Forest fires”[TIAB] OR “Wild-land fire”[TIAB] OR “Wild-land fires”[TIAB] |  | 3,956 |
| 8 | Displacement*[TIAB] OR Migration*[TIAB] OR Migrant*[TIAB] |  | 412,015 |
| 9 | “Mental health”[TIAB] OR “Mental disorder”[TIAB]  OR “Post-traumatic stress disorder”[TIAB]  OR Anxiet*[TIAB]  OR Depress*[TIAB]  OR Solastalgia[TIAB]  OR Ecoanxi* OR eco-anxi*[TIAB] OR Ecoguilt*[TIAB]  OR “Ecological grief””[TIAB]  OR “Biospheric concern”[TIAB] |  | 760,537 |
| 12 | South-America*[TIAB] OR South America*[TIAB] OR Argentin*[TIAB] OR Bolivia*[TIAB] OR Brazil*[TIAB] OR Chile*[TIAB] OR Colombia*[TIAB] OR Ecuador*[TIAB] OR Paraguay*[TIAB] OR Peru*[TIAB] OR Uruguay*[TIAB] OR Venezuela*[TIAB] OR Ande*[TIAB] OR Amazon[TIAB] OR Altiplano[TIAB] OR Surinam*[TIAB] OR Guyan*[TIAB] |  | 251,703 |
| 13 | #2 OR #3 OR #4 OR #5 OR #6 OR #7 |  | 443,940 |
| 14 | #1 AND #13 AND (#8 OR #9) AND #12 | Humans | 35 |
| **Database: Pubmed MeSH** | | | |
| **Date: 24 October 2021** | | | |
| **Search** | **Strategy** | **Filters (if any)** | **Results** |
| 1 | ("Global Warming"[Mesh]) OR "Climate Change"[Mesh] |  | 23,906 |
| 2 | ((((("Extreme Heat"[Mesh]) OR "Cold Temperature"[Mesh]) OR "Hot Temperature"[Mesh]) OR "Extreme Hot Weather"[Mesh]) OR "Extreme Cold Weather"[Mesh]) OR "Extreme Weather"[Mesh] |  | 190,930 |
| 3 | (((("Rain"[Mesh]) OR "Humidity"[Mesh]) OR "Droughts"[Mesh]) OR "Floods"[Mesh]) OR "Water Insecurity"[Mesh] |  | 40,864 |
| 4 | (("Extreme Weather"[Mesh]) OR "Cyclonic Storms"[Mesh]) OR "Tornadoes"[Mesh] |  | 2,982 |
| 5 | (("El Nino-Southern Oscillation"[Mesh]) OR "Sea Level Rise"[Mesh]) OR "Harmful Algal Bloom"[Mesh] |  | 1,880 |
| 6 | ("Biodiversity"[Mesh]) OR "Extinction, Biological"[Mesh] |  | 104,059 |
| 7 | "Wildfires"[Mesh] |  | 702 |
| 8 | “Human Migration”[Mesh] |  | 27,043 |
| 9 | (((((((((((((((((("Mental Health"[Mesh]) OR "Mental Disorders"[Mesh]) OR "Stress Disorders, Post-Traumatic"[Mesh]) OR "Anxiety"[Mesh]) OR "Depression"[Mesh]) OR "Stress Disorders, Traumatic"[Mesh]) OR "Stress, Psychological"[Mesh]) OR "Grief"[Mesh]) OR "Psychological Distress"[Mesh]) OR "Psychological Trauma"[Mesh]) OR "Obsessive-Compulsive Disorder"[Mesh]) OR "Panic"[Mesh]) OR "Psychotic Disorders"[Mesh]) OR "Delusions"[Mesh]) OR "Mania"[Mesh]) OR "Phobic Disorders"[Mesh]) OR "Somatoform Disorders"[Mesh]) OR "Suicide"[Mesh]) OR "Dementia"[Mesh] |  | 1,620,582 |
| 10 | (((((((((((("South America"[Mesh]) OR "Argentina"[Mesh]) OR "Bolivia"[Mesh]) OR "Brazil"[Mesh]) OR "Chile"[Mesh]) OR "Colombia"[Mesh]) OR "Ecuador"[Mesh]) OR "Paraguay"[Mesh]) OR "Peru"[Mesh]) OR "Suriname"[Mesh]) OR "Uruguay"[Mesh]) OR "Venezuela"[Mesh]) OR "Guyana"[Mesh] |  | 180,741 |
| 11 | #2 OR #3 OR #4 OR #5 OR #6 OR #7 |  | 335,061 |
| 13 | #1 AND #11 AND (#8 OR #9) AND #10 | Humans | 92 |
| **Database: ProQuest** | | | |
| **Date: 24 October 2021** | | | |
| **Search** | **Strategy** | **Filters (if any)** | **Results** |
| 1 | ab("Climat* change$" OR "Climat* disrupt*" OR "Climat* hazard$" OR "Climat* extreme*" OR "Climat* catastroph*" OR "Climat* shift*" OR "Global warming" OR "Climat* variation$" OR "Climat* variab*" OR "Chang* climat*" OR "Weather change*" OR "Meteorol* chang*" OR "Temperature$ chang*" OR "Temperature* extreme$" OR "Temperature* anomal*" OR "Rising temperature*" OR "Temperature increase" OR "Temperature decrease" OR heat-wave OR Warm-spell OR Cold-spell OR "Extreme heat" OR "Extreme cold" OR "High temperature$" OR "Low temperature$" OR "Precipitation$" OR "Humid* chang*" OR Mold OR Moist OR Evaporation OR Muggy OR Drought OR "Water scarcity" OR "Water stress" OR "Aridity" OR Dry-spell OR "Water Shortage$" OR "Palmer Drought Severity Index" OR Flood OR Inundation OR Deluge OR Downpour* OR Rain* OR Alluvion OR "Extreme weather event$" OR "Extreme climat* event$" OR "Extreme hydrometeorological event$" OR "Extreme meteorological event$" OR Hurricane OR Tornado OR Typhoon OR Storm OR Windstorm OR "Tropical cyclone$" OR "Ocean acidification" OR "Sea acidification" OR "Ocean warming" OR "Ocean heat*" OR "Sea heat*" OR "Sea warming" OR "El Ni$o" OR "Sea surface temperature" OR ENSO OR " Southern Oscillation" OR "Sea level ris*" OR "Rising sea level*" OR "Coastal erosion" OR "Algae bloom*" OR "Brown tide" OR "Red tide" OR "Marine bloom" OR "Water bloom" OR "Black water" OR "Retreat of glacier*" OR "Melting sea ice" OR "Glacial meltwater*" OR "Glacier recession" OR "Reduced sea ice" OR "Ice sheet melt*" OR "Glacier shrink*" OR biodiversity OR "Biodiversity change*" OR "Biodiversity loss*" OR "Specie* extinction" OR "Specie* loss" OR "Specie* disappearance" OR "Biological diversity loss*" OR "Ecosystem change*" OR "Vector* suitability" OR "Vector* adequacy" OR "Vector* capacity" OR Wild-fire OR Bush-fire OR "Forest fire$" OR "Wild-land fire*") AND ab(health OR well-being OR quality-of-life OR welfare OR Death$ OR Mortal* OR Fatalit* OR Casualt* OR Disease$ OR Disorder$ OR Illness* OR Sickness* OR Indisposition$ OR Unhealth* OR Injur*) AND ab(South-America* OR Argentin* OR Bolivia* OR Brazil* OR Chile* OR Colombia* OR Ecuador* OR Paraguay* OR Peru* OR Uruguay* OR Venezuela* OR Andes OR Amazon OR Altiplano OR Surinam* OR Guyan*) AND ab(“Mental health*” OR “Mental disorder*”  OR “Post-traumatic stress disorder”  OR Anxiet*  OR Depress*  OR Solastalgia  OR Ecoanxi* OR eco-anxi* OR Ecoguilt*  OR “Ecological grief” OR “Biospheric concern” OR Displacement$ OR Migration$ OR Migrant$) |  | 96 |
| **Database: Scopus** | | | |
| **Date: 24 October 2021** | | | |
| **Search** | **Strategy** | **Filters (if any)** | **Results** |
|  | ( TITLE-ABS-KEY ( {Climate change} OR {Climate disruption} OR {Climate hazard} OR {Climate extreme} OR {Climate catastrophe} OR {Climate shift} OR {Global warming} OR {Climate variation} OR {Climate variability} OR {Changing climate} OR {Weather change} OR {Meteorological change} OR {Temperature change} OR {Temperature extreme} OR {Temperature anomaly} OR {Rising temperature} OR {Temperature increase} OR {Temperature decrease} OR heat-wave OR warm-spell OR cold-spell OR {Extreme heat} OR {Extreme cold} OR {High temperature} OR {Low temperature} OR precipitation* OR {Humidity change} OR mold OR moist OR evaporation OR muggy OR drought* OR {Water scarcity} OR {Water stress} OR aridity OR dry-spell OR {Water Shortage} OR {Palmer Drought Severity Index} OR flood* OR inundation* OR deluge OR downpour* OR rain* OR alluvion* OR {Extreme weather event} OR {Extreme climate event} OR {Extreme hydrometeorological event} OR {Extreme meteorological event} OR hurricane* OR tornado* OR typhoon* OR storm* OR windstorm* OR {Tropical cyclone} OR {ocean acidification} OR {sea acidification} OR {ocean warming} OR {ocean heating} OR {sea heating} OR {sea warming} OR {el nino} OR {el niño} OR {sea surface temperature} OR enso OR {southern oscillation} OR {sea level rise} OR {rising sea level} OR {coastal erosion} OR {algae bloom} OR {brown tide} OR {red tide} OR {marine bloom} OR {water bloom} OR {black water} OR {retreat of glacier} OR {melting sea ice} OR {glacial meltwater} OR {glacier recession} OR {reduced sea ice} OR {ice sheet melting} OR {glacier shrink} OR biodiversity OR {Biodiversity change} OR {Biodiversity loss} OR {Specie extinction} OR {Specie loss} OR {Specie disappearance} OR {Biological diversity loss} OR {Ecosystem change} OR {Vector suitability} OR {Vector adequacy} OR {Vector capacity} OR wild-fire OR wildfire OR bush-fire OR bushfire OR {Forest fire} OR {Wild-land fire} ) AND TITLE-ABS-KEY ( "South-America*" OR argentin* OR bolivia* OR brazil* OR chile* OR colombia* OR ecuador* OR paraguay* OR peru* OR uruguay* OR venezuela* OR andes OR amazon OR altiplano OR surinam* OR guyan* ) AND TITLE-ABS-KEY ( {Mental health} OR {Mental disorder} OR {Post-traumatic stress disorder} OR anxiet* OR depress* OR solastalgia OR ecoanxi* OR eco-anxi* OR ecoguilt* OR {Ecological grief} OR {Biospheric concern} ) ) AND ( LIMIT-TO ( SUBJAREA , "ENVI" ) OR LIMIT-TO ( SUBJAREA , "MEDI" ) OR LIMIT-TO ( SUBJAREA , "PSYC" ) OR LIMIT-TO ( SUBJAREA , "NURS" ) OR LIMIT-TO ( SUBJAREA , "HEAL" ) ) AND ( LIMIT-TO ( EXACTKEYWORD , "Human" ) OR LIMIT-TO ( EXACTKEYWORD , "Humans" )) |  | 42 |
| **Database: SciELO** | | | |
| **Date: 24 October 2021** | | | |
| **Search** | **Strategy** | **Filters (if any)** | **Results** |
| 1 | ((("climate change" OR "climate disruption" OR "climate hazard" OR "climate extreme" OR "climate catastrophe" OR "climate shift" OR "global warming" OR "climate variation" OR "climate variability" OR "changing climate" OR "weather change" OR "meteorological change") OR ("temperature change" OR "temperature extreme" OR "temperature anomaly" OR "rising temperature" OR "temperature increase" OR "temperature decrease" OR heat-wave OR warm-spell OR cold-spell OR "extreme heat" OR "extreme cold" OR "high temperature" OR "low temperature") OR (precipitation OR "humidity change" OR mold OR moist OR evaporation OR muggy OR drought OR "water scarcity" OR "water stress" OR aridity OR dry-spell OR "water shortage" OR "palmer drought severity index" OR flood OR inundation OR deluge OR downpour OR rain OR alluvion) OR ("extreme weather event" OR "extreme climate event" OR "extreme meteorological event" OR "extreme hydrometeorological event" OR hurricane OR tornado OR typhoon OR storm OR windstorm OR "tropical cyclone") OR ("ocean acidification" OR "sea acidification" OR "ocean warming" OR "ocean heating" OR "sea heating" OR "sea warming" OR "el nino" OR "el niño" OR "sea surface temperature" OR enso OR "southern oscillation" OR "sea level rise" OR "rising sea level" OR "coastal erosion" OR "algae bloom" OR "brown tide" OR "red tide" OR "marine bloom" OR "water bloom" OR "black water" OR "retreat of glacier" OR "melting sea ice" OR "glacial meltwater" OR "glacier recession" OR "reduced sea ice" OR "ice sheet melting" OR "glacier shrink") OR (biodiversity OR "biodiversity change" OR "biodiversity loss" OR "specie extinction" OR "specie loss" OR "specie disappearance" OR "biological diversity loss" OR "ecosystem change" OR "vector suitability" OR "vector adequacy" OR "vector capacity") OR (wild-fire OR wildfire OR bush-fire OR bushfire OR "forest fire" OR "wild land fire")) AND (“mental health” OR “mental disorder” OR “post-traumatic stress disorder” OR anxiety* OR depress* OR solastagia OR ecoanxi* OR eco-anxi* OR ecoguilt OR “ecological grief” OR “biospheric concern”) AND ("South America" OR South-America* OR argentin* OR bolivia* OR brazil* OR chile* OR colombia* OR ecuador* OR paraguay* OR peru* OR uruguay* OR venezuela* OR andes OR amazon OR altiplano OR surinam* OR guyan*)) AND (human) |  | 193 |
| **Database: BIREME/LILACS** | | | |
| **Date: 24 October 2021** | | | |
| **Search** | **Strategy** | **Filters (if any)** | **Results** |
|  | (ab:(("climate change") OR ("climate disruption") OR ("climate hazard") OR ("climate extreme") OR ("climate catastrophe") OR ("climate shift") OR ("global warming") OR ("climate variation") OR ("climate variability") OR ("changing climate") OR ("weather change") OR ("meteorological change") OR ("temperature change") OR ("temperature extreme") OR ("temperature anomaly") OR ("rising temperature") OR ("temperature increase") OR ("temperature decrease") OR (heat-wave) OR (warm-spell) OR (cold-spell) OR ("extreme heat") OR ("extreme cold") OR ("high temperature") OR ("low temperature") OR (precipitation) OR ("humidity change") OR (mold) OR (moist) OR (evaporation) OR (muggy) OR (drought) OR ("water scarcity") OR ("water stress") OR (aridity) OR (dry-spell) OR ("water shortage") OR ("palmer drought severity index") OR (flood) OR (inundation) OR (deluge) OR (downpour) OR (rain) OR (alluvion) OR ("extreme weather event") OR ("extreme climate event") OR ("extreme meteorological event") OR ("extreme hydrometeorological event") OR (hurricane) OR (tornado) OR (typhoon) OR (storm) OR (windstorm) OR ("tropical cyclone") OR ("ocean acidification") OR ("sea acidification") OR ("ocean warming") OR ("ocean heating") OR ("sea heating") OR ("sea warming") OR ("el nino") OR ("el niño") OR ("sea surface temperature") OR (enso) OR ("southern oscillation") OR ("sea level rise") OR ("rising sea level") OR ("coastal erosion") OR ("algae bloom") OR ("brown tide") OR ("red tide") OR ("marine bloom") OR ("water bloom") OR ("black water") OR ("retreat of glacier") OR ("melting sea ice") OR ("glacial meltwater") OR ("glacier recession") OR ("reduced sea ice") OR ("ice sheet melting") OR ("glacier shrink") OR (biodiversity) OR ("biodiversity change") OR ("biodiversity loss") OR ("specie extintion") OR ("specie loss") OR ("specie disappearance") OR ("biological diversity loss") OR ("ecosystem change") OR ("vector suitability") OR ("vector adequacy") OR ("vector capacity") OR (wild-fire) OR (wildfire) OR (bush-fire) OR (bushfire) OR ("forest fire") OR ("wild land fire"))) AND (ab:(("mental health") OR ("mental disorder") OR ("post-traumatic stress disorder") OR (anxiety) OR (depress) OR (solastalgia) OR (ecoanxiety) OR (eco-anxiety) OR (ecoguilty) OR ("ecological grief") OR ("biospheric concern") OR (Displacement) OR (Migration) OR (Migrant))) AND (ab:(("South America") OR (South-America) OR (argentina) OR (bolivia) OR (brazil) OR (chile) OR (colombia) OR (ecuador) OR (paraguay) OR (peru) OR (uruguay) OR (venezuela) OR (andes) OR (amazon) OR (altiplano) OR (suriname) OR (guyana))) |  | 39 |

**Table S3.** Search strategies for: *To what extent human populations in South America are exposed to the hazards of climate change?*

| **Database: Web of Science** | | | |
| --- | --- | --- | --- |
| **Date: 10 October 2021** | | | |
| **Search** | **Strategy** | **Filters (if any)** | **Results** |
| 1 | TS=(“Climat* change$” OR “Climat* disrupt*” OR “Climat* hazard$” OR “Climat* extreme*” OR “Climat* catastroph*” OR “Climat* shift*” OR “Global warming” OR “Climat* variation$” OR “Climat* variab*” OR “Chang* climat*” OR “Weather change*” OR “Meteorol* chang*”) | None | 382,445 |
| 2 | TS=(“Temperature$ chang*” OR “Temperature* extreme$” OR “Temperature* anomal*” OR “Rising temperature*” OR “Temperature increase” OR “Temperature decrease” OR heat-wave OR Warm-spell OR Cold-spell OR “Extreme heat” OR “Extreme cold” OR “High temperature$” OR “Low temperature$”) | None | 841,421 |
| 3 | TS=(“Precipitation$” OR Moist OR Muggy OR Drought$ OR “Water scarcity” OR “Water stress” OR “Aridity” OR Dry-spell OR “Water Shortage$” OR “Palmer Drought Severity Index” OR Flood$ OR Inundation$ OR Deluge OR Downpour* OR Rain* OR Alluvion$) | None | 1,209,999 |
| 4 | TS=(“Extreme weather event$” OR “Extreme climat* event$” OR “Extreme hydrometeorological event$” OR “Extreme meteorological event$” OR Hurricane$ OR Tornado$ OR Typhoon$ OR Storm$ OR Windstorm$ OR “Tropical cyclone$”) | None | 139,900 |
| 5 | TS=(“Ocean acidification” OR “Sea acidification” OR “Ocean warming” OR “Ocean heat*” OR “El Ni$o” OR “Sea surface temperature” OR ENSO OR “ Southern Oscillation” OR “Sea level ris*” OR “Rising sea level*” OR “Coastal erosion” OR “Algae bloom*” OR “Brown tide” OR “Red tide” OR “Marine bloom” OR “Water bloom” OR “Black water” OR “Retreat of glacier*” OR “Melting sea ice” OR “Glacial meltwater*” OR “Glacier recession” OR “Reduced sea ice” OR “Ice sheet melt*” OR “Glacier shrink*” ) | None | 95,738 |
| 6 | TS=(biodiversity OR “Biodiversity change*” OR “Biodiversity loss*” OR “Specie* extinction” OR “Specie* loss” OR “Specie* disappearance” OR “Biological diversity loss*” OR “Ecosystem change*” OR “Vector* suitability” OR “Vector* adequacy” OR “Vector* capacity”) | None | 167,135 |
| 7 | TS=(Wild-fire$ OR Bush-fire$ OR “Forest fire$” OR “Wild-land fire*”) | None | 12,716 |
| 8 | TS=(health OR well-being OR quality-of-life OR welfare) | None | 3,156,161 |
| 9 | TS=(Death$ OR Mortal* OR Fatalit* OR Casualt* OR Disease$ OR Disorder$ OR Illness* OR Sickness* OR Indisposition$ OR Unhealth* OR Injur*) | None | 8,235,107 |
| 10 | TS=(Heat OR Heat-stroke$ OR Faintness OR “Warm skin” OR Dehydration OR Heat-stress OR “Heat exhaustion” OR “Heat hyperpyrexia” OR “Heat-related death$” OR “Respiratory disease$” OR “Vector-borne disease$” OR “Mosquito-borne Disease$” OR “Rodent-borne disease$” OR “Food-borne disease$” OR “Water-borne disease$” OR Poisoning) | None | 1,714,038 |
| 11 | TS=(“Food secur*” OR “Food scarcity” OR “Food shortage” OR “Food deprivation” OR “Food deficit” OR “Food shortfall” OR Malnutrit* OR Undernutri* OR Undernourish* OR Malnourish* OR Starv* OR Underfed OR Famine OR Hunger) | None | 184,790 |
| 12 | TS=(Exposure OR Exposition OR Exposed) | None | 1,813,187 |
| 13 | TS=(South-America* OR Argentin* OR Bolivia* OR Brazil* OR Chile* OR Colombia* OR Ecuador* OR Paraguay* OR Peru* OR Uruguay* OR Venezuela* OR Andes OR Amazon OR Altiplano OR Surinam* OR Guyan*) | None | 702,052 |
| 14 | TS=(human) | None | - |
| 15 | (#1 OR #2 OR #3 OR #4 OR #5 OR #6 OR #7) AND (#8 OR #9 OR #10 OR #11) AND #12 AND #13 AND 14 |  | 276 |
| **Database: PubMed** | | | |
| **Date: 24 October 2021** | | | |
| **Search** | **Strategy** | **Filters (if any)** | **Results** |
| 1 | “Climate change”[TIAB] OR “Climate changes”[TIAB] OR “Climatic change”[TIAB] OR “Climatic changes”[TIAB] OR “Climate disruption”[TIAB] OR “Climate disruptions”[TIAB] OR “Climatic disruption”[TIAB] OR “Climatic disruptions”[TIAB] OR “Climate hazard”[TIAB] OR “Climate hazards”[TIAB] OR “Climatic hazard”[TIAB] OR “Climatic hazards”[TIAB] OR “Climate extreme”[TIAB] OR “Climate extremes”[TIAB] OR “Climatic extreme”[TIAB] OR “Climatic extremes”[TIAB] OR “Climate catastrophe”[TIAB] OR “Climate catastrophes”[TIAB] OR “Climatic catastrophe”[TIAB] OR “Climatic catastrophes”[TIAB] OR “Climate shift”[TIAB] OR “Climate shifts”[TIAB] OR “Climatic shift”[TIAB] OR “Climatic shifts”[TIAB] OR “Global warming”[TIAB] OR “Climate variation”[TIAB] OR “Climate variations”[TIAB] OR “Climatic variation”[TIAB] OR “Climatic variations”[TIAB] OR “Climate variability”[TIAB] OR “Climate variabilities”[TIAB] OR “Climatic variability”[TIAB] OR “Climatic variabilities”[TIAB] OR “Changing Climate”[TIAB] OR “Weather change”[TIAB] OR “Weather changes”[TIAB] OR “Meteorological change”[TIAB] |  | 57,543 |
| 2 | “Temperature changes”[TIAB] OR “Temperature change”[TIAB] OR “Temperature changing”[TIAB] OR “Temperature extreme”[TIAB] OR “Temperature extremes”[TIAB] OR “Temperature anomaly”[TIAB] OR “Temperature anomalies”[TIAB] OR “Rising temperature”[TIAB] OR “Rising temperatures”[TIAB] OR “Temperature increase”[TIAB] OR “Temperature increasing”[TIAB] OR “Temperature decrease”[TIAB] OR “Temperature decreasing”[TIAB] OR heat-wave[TIAB] OR heatwave[TIAB] OR “heat wave”[TIAB] OR warm spell[TIAB] OR warm-spell[TIAB] OR “Warm spell”[TIAB] OR “Warm spells”[TIAB] OR cold spell[TIAB] OR cold-spell[TIAB] OR “Cold spell”[TIAB] OR “Cold spells”[TIAB] OR “Extreme heat”[TIAB] OR “Extreme cold”[TIAB] OR “High temperature”[TIAB] OR “High temperatures”[TIAB] OR “Higher temperatures”[TIAB] OR “Higher temperature”[TIAB] OR “Low temperature”[TIAB] OR “Low temperatures”[TIAB] OR “Lower temperatures”[TIAB] OR “Lower temperature”[TIAB] |  | 143,297 |
| 3 | Precipitati*[TIAB] OR Moist*[TIAB] OR Evaporation[TIAB] OR Muggy[TIAB] OR Drought*[TIAB] OR “Water scarcity”[TIAB] OR “Water stress”[TIAB] OR “Aridity”[TIAB] OR dry-spell[TIAB] OR “Dry spell”[TIAB] OR “Water Shortage”[TIAB] OR “Palmer Drought Severity Index”[TIAB] OR Flood*[TIAB] OR Inundation*[TIAB] OR Deluge[TIAB] OR Downpour*[TIAB] OR Rain*[TIAB] OR Alluvion*[TIAB] |  | 247,308 |
| 4 | “Extreme weather event”[TIAB] OR “Extreme weather events”[TIAB] OR “Extreme climate event”[TIAB] OR “Extreme climatic event”[TIAB] OR “Extreme climate events”[TIAB] OR “Extreme climate event”[TIAB] OR “Extreme hydrometeorological event”[TIAB] OR “Extreme meteorological events”[TIAB] OR Hurricane*[TIAB] OR Tornado*[TIAB] OR Typhoon*[TIAB] OR Storm*[TIAB] OR Windstorm*[TIAB] OR “Tropical cyclone”[TIAB] OR “Tropical cyclones”[TIAB] |  | 25,122 |
| 5 | “Ocean acidification”[TIAB] OR “Ocean warming”[TIAB] OR “Ocean heat”[TIAB] OR “Ocean heating”[TIAB] OR “El Nino”[TIAB] OR “El Niño”[TIAB] OR “Sea surface temperature”[TIAB] OR ENSO[TIAB] OR “Southern Oscillation”[TIAB] OR “Sea level rise”[TIAB] OR “Sea level rising”[TIAB] OR “Rising sea level”[TIAB] OR “Rising sea levels”[TIAB] OR “Coastal erosion”[TIAB] OR “Algae bloom”[TIAB] OR “Brown tide”[TIAB] OR “Red tide”[TIAB] OR “Marine bloom”[TIAB] OR “Water bloom”[TIAB] OR “Black water”[TIAB] OR “Retreat of glacier”[TIAB] OR “Retreat of glaciers”[TIAB] OR “Melting sea ice”[TIAB] OR “Glacial meltwater”[TIAB] OR “Glacier recession”[TIAB] OR “Reduced sea ice”[TIAB] OR “Ice sheet melt”[TIAB] OR “Ice sheet melting”[TIAB] OR “Glacier shrinking”[TIAB] OR “Glacier shrinkage”[TIAB] |  | 9,203 |
| 6 | biodiversity[TIAB] OR “Biodiversity change”[TIAB] OR “Biodiversity changes”[TIAB] OR “Biodiversity loss”[TIAB] OR “Specie extinction”[TIAB] OR “Species extinction”[TIAB] OR “Specie extinctions”[TIAB] OR “Species extinctions”[TIAB] OR “Specie loss”[TIAB] OR “Species loss”[TIAB] OR “Specie disappearance”[TIAB] OR “Species disappearance”[TIAB] OR “Biological diversity loss” [TIAB] OR “Ecosystem change”[TIAB] OR “Ecosystem changes”[TIAB] OR “Vector suitability”[TIAB] OR “Vector adequacy”[TIAB] OR “Vector capacity”[TIAB] OR “vectorial suitability”[TIAB] OR “vectorial adequacy”[TIAB] OR “vectorial capacity”[TIAB] |  | 35,054 |
| 7 | “Wild fire”[TIAB] OR “Wild fires”[TIAB] OR wild-fire*[TIAB] OR Wildfire*[TIAB] OR “Bush fire”[TIAB] OR “Bush fires”[TIAB] OR bushfire*[TIAB] OR bush-fire*[TIAB] OR “Forest fire”[TIAB] OR “Forest fires”[TIAB] OR “Wild-land fire”[TIAB] OR “Wild-land fires”[TIAB] |  | 3,956 |
| 8 | South-America*[TIAB] OR South America*[TIAB] OR Argentin*[TIAB] OR Bolivia*[TIAB] OR Brazil*[TIAB] OR Chile*[TIAB] OR Colombia*[TIAB] OR Ecuador*[TIAB] OR Paraguay*[TIAB] OR Peru*[TIAB] OR Uruguay*[TIAB] OR Venezuela*[TIAB] OR Ande*[TIAB] OR Amazon[TIAB] OR Altiplano[TIAB] OR Surinam*[TIAB] OR Guyan*[TIAB] |  | 251,703 |
| 9 | Exposure[TIAB] OR Exposition[TIAB] OR Exposed[TIAB] |  | 1,218,310 |
| 10 | #2 OR #3 OR #4 OR #5 OR #6 OR #7 |  | 443,940 |
| 11 | #1 AND #8 AND #9 AND #10 | Humans | 218 |
| **Database: Pubmed MeSH** | | | |
| **Date: 24 October 2021** | | | |
| **Search** | **Strategy** | **Filters (if any)** | **Results** |
| 1 | ("Global Warming"[Mesh]) OR "Climate Change"[Mesh] |  | 23,906 |
| 2 | ((((("Extreme Heat"[Mesh]) OR "Cold Temperature"[Mesh]) OR "Hot Temperature"[Mesh]) OR "Extreme Hot Weather"[Mesh]) OR "Extreme Cold Weather"[Mesh]) OR "Extreme Weather"[Mesh] |  | 190,930 |
| 3 | (((("Rain"[Mesh]) OR "Humidity"[Mesh]) OR "Droughts"[Mesh]) OR "Floods"[Mesh]) OR "Water Insecurity"[Mesh] |  | 40,864 |
| 4 | (("Extreme Weather"[Mesh]) OR "Cyclonic Storms"[Mesh]) OR "Tornadoes"[Mesh] |  | 2,982 |
| 5 | (("El Nino-Southern Oscillation"[Mesh]) OR "Sea Level Rise"[Mesh]) OR "Harmful Algal Bloom"[Mesh] |  | 1,880 |
| 6 | ("Biodiversity"[Mesh]) OR "Extinction, Biological"[Mesh] |  | 104,059 |
| 7 | "Wildfires"[Mesh] |  | 702 |
| 8 | "Environmental Exposure"[Mesh] |  | 324,377 |
| 9 | (((((((((((("South America"[Mesh]) OR "Argentina"[Mesh]) OR "Bolivia"[Mesh]) OR "Brazil"[Mesh]) OR "Chile"[Mesh]) OR "Colombia"[Mesh]) OR "Ecuador"[Mesh]) OR "Paraguay"[Mesh]) OR "Peru"[Mesh]) OR "Suriname"[Mesh]) OR "Uruguay"[Mesh]) OR "Venezuela"[Mesh]) OR "Guyana"[Mesh] |  | 180,741 |
| 10 | #2 OR #3 OR #4 OR #5 OR #6 OR #7 |  | 335,061 |
| 11 | #1 AND #8 AND #9 AND #10 | Humans | 36 |
| **Database: ProQuest** | | | |
| **Date: 24 October 2021** | | | |
| **Search** | **Strategy** | **Filters (if any)** | **Results** |
|  | ab("Climat* change$" OR "Climat* disrupt*" OR "Climat* hazard$" OR "Climat* extreme*" OR "Climat* catastroph*" OR "Climat* shift*" OR "Global warming" OR "Climat* variation$" OR "Climat* variab*" OR "Chang* climat*" OR ("weather change" OR "weather changed" OR "weather changes") OR "Meteorol* chang*" OR "Temperature$ chang*" OR "Temperature* extreme$" OR "Temperature* anomal*" OR ("rising temperature" OR "rising temperatures") OR "Temperature increase" OR "Temperature decrease" OR heat-wave OR Warm-spell OR Cold-spell OR "Extreme heat" OR "Extreme cold" OR "High temperature$" OR "Low temperature$" OR "Precipitation$" OR "Humid* chang*" OR Mold OR Moist OR Evaporation OR Muggy OR Drought OR "Water scarcity" OR "Water stress" OR "Aridity" OR Dry-spell OR "Water Shortage$" OR "Palmer Drought Severity Index" OR Flood OR Inundation OR Deluge OR Downpour* OR Rain* OR Alluvion OR "Extreme weather event$" OR "Extreme climat* event$" OR "Extreme hydrometeorological event$" OR "Extreme meteorological event$" OR Hurricane OR Tornado OR Typhoon OR Storm OR Windstorm OR "Tropical cyclone$" OR "Ocean acidification" OR "Sea acidification" OR "Ocean warming" OR ("ocean heat") OR "Sea heat*" OR "Sea warming" OR "El Ni$o" OR "Sea surface temperature" OR ENSO OR " Southern Oscillation" OR "Sea level ris*" OR "Rising sea level*" OR "Coastal erosion" OR ("algae bloom" OR "algae blooms") OR "Brown tide" OR "Red tide" OR "Marine bloom" OR "Water bloom" OR "Black water" OR "Retreat of glacier*" OR "Melting sea ice" OR ("glacial meltwater" OR "glacial meltwaters") OR "Glacier recession" OR "Reduced sea ice" OR "Ice sheet melt*" OR "Glacier shrink*" OR biodiversity OR "Biodiversity change*" OR ("biodiversity loss") OR "Specie* extinction" OR "Specie* loss" OR "Specie* disappearance" OR "Biological diversity loss*" OR ("ecosystem change" OR "ecosystem changes") OR "Vector* suitability" OR "Vector* adequacy" OR "Vector* capacity" OR Wild-fire OR Bush-fire OR "Forest fire$" OR "Wild-land fire*") AND ab(Exposure OR Exposition OR Exposed) AND ab(South-America* OR Argentin* OR Bolivia* OR Brazil* OR Chile* OR Colombia* OR Ecuador* OR Paraguay* OR Peru* OR Uruguay* OR Venezuela* OR Andes OR Amazon OR Altiplano OR Surinam* OR Guyan*) AND ab(human) |  | 124 |
| **Database: Scopus** | | | |
| **Date: 24 October 2021** | | | |
| **Search** | **Strategy** | **Filters (if any)** | **Results** |
|  | ( TITLE-ABS-KEY ( {Climate change} OR {Climate disruption} OR {Climate hazard} OR {Climate extreme} OR {Climate catastrophe} OR {Climate shift} OR {Global warming} OR {Climate variation} OR {Climate variability} OR {Changing climate} OR {Weather change} OR {Meteorological change} OR {Temperature change} OR {Temperature extreme} OR {Temperature anomaly} OR {Rising temperature} OR {Temperature increase} OR {Temperature decrease} OR heat-wave OR warm-spell OR cold-spell OR {Extreme heat} OR {Extreme cold} OR {High temperature} OR {Low temperature} OR precipitation* OR {Humidity change} OR mold OR moist OR evaporation OR muggy OR drought* OR {Water scarcity} OR {Water stress} OR aridity OR dry-spell OR {Water Shortage} OR {Palmer Drought Severity Index} OR flood* OR inundation* OR deluge OR downpour* OR rain* OR alluvion* OR {Extreme weather event} OR {Extreme climate event} OR {Extreme hydrometeorological event} OR {Extreme meteorological event} OR hurricane* OR tornado* OR typhoon* OR storm* OR windstorm* OR {Tropical cyclone} OR {ocean acidification} OR {sea acidification} OR {ocean warming} OR {ocean heating} OR {sea heating} OR {sea warming} OR {el nino} OR {el niño} OR {sea surface temperature} OR enso OR {southern oscillation} OR {sea level rise} {rising sea level} OR {coastal erosion} OR {algae bloom} OR {brown tide} OR {red tide} OR {marine bloom} OR {water bloom} OR {black water} OR {retreat of glacier} OR {melting sea ice} OR {glacial meltwater} OR {glacier recession} OR {reduced sea ice} OR {ice sheet melting} OR {glacier shrink} OR biodiversity OR {Biodiversity change} OR {Biodiversity loss} OR {Specie extinction} OR {Specie loss} OR {Specie disappearance} OR {Biological diversity loss} OR {Ecosystem change} OR {Vector suitability} OR {Vector adequacy} OR {Vector capacity} OR wild-fire OR wildfire OR bush-fire OR bushfire OR {Forest fire} OR {Wild-land fire} ) AND TITLE-ABS-KEY ( "South-America*" OR argentin* OR bolivia* OR brazil* OR chile* OR colombia* OR ecuador* OR paraguay* OR peru* OR uruguay* OR venezuela* OR andes OR amazon OR altiplano OR surinam* OR guyan* ) AND TITLE-ABS-KEY ( exposure OR exposition OR exposed ) ) AND ( LIMIT-TO ( SUBJAREA , "ENVI" ) OR LIMIT-TO ( SUBJAREA , "MEDI" ) OR LIMIT-TO ( SUBJAREA , "NURS" ) OR LIMIT-TO ( SUBJAREA , "HEAL" ) OR LIMIT-TO ( SUBJAREA , "PSYC" ) ) AND ( LIMIT-TO ( EXACTKEYWORD , "Human" ) ) |  | 376 |
| **Database: SciELO** | | | |
| **Date: 24 October 2021** | | | |
| **Search** | **Strategy** | **Filters (if any)** | **Results** |
| 1 | ((("climate change" OR "climate disruption" OR "climate hazard" OR "climate extreme" OR "climate catastrophe" OR "climate shift" OR "global warming" OR "climate variation" OR "climate variability" OR "changing climate" OR "weather change" OR "meteorological change") OR ("temperature change" OR "temperature extreme" OR "temperature anomaly" OR "rising temperature" OR "temperature increase" OR "temperature decrease" OR heat-wave OR warm-spell OR cold-spell OR "extreme heat" OR "extreme cold" OR "high temperature" OR "low temperature") OR (precipitation OR "humidity change" OR mold OR moist OR evaporation OR muggy OR drought OR "water scarcity" OR "water stress" OR aridity OR dry-spell OR "water shortage" OR "palmer drought severity index" OR flood OR inundation OR deluge OR downpour OR rain OR alluvion) OR ("extreme weather event" OR "extreme climate event" OR "extreme meteorological event" OR "extreme hydrometeorological event" OR hurricane OR tornado OR typhoon OR storm OR windstorm OR "tropical cyclone") OR ("ocean acidification" OR "sea acidification" OR "ocean warming" OR "ocean heating" OR "sea heating" OR "sea warming" OR "el nino" OR "el niño" OR "sea surface temperature" OR enso OR "southern oscillation" OR "sea level rise" OR "rising sea level" OR "coastal erosion" OR "algae bloom" OR "brown tide" OR "red tide" OR "marine bloom" OR "water bloom" OR "black water" OR "retreat of glacier" OR "melting sea ice" OR "glacial meltwater" OR "glacier recession" OR "reduced sea ice" OR "ice sheet melting" OR "glacier shrink") OR (biodiversity OR "biodiversity change" OR "biodiversity loss" OR "specie extinction" OR "specie loss" OR "specie disappearance" OR "biological diversity loss" OR "ecosystem change" OR "vector suitability" OR "vector adequacy" OR "vector capacity") OR (wild-fire OR wildfire OR bush-fire OR bushfire OR "forest fire" OR "wild land fire")) AND (exposure OR exposition OR exposed) AND ("South America" OR South-America* OR argentin* OR bolivia* OR brazil* OR chile* OR colombia* OR ecuador* OR paraguay* OR peru* OR uruguay* OR venezuela* OR andes OR amazon OR altiplano OR surinam* OR guyan*)) AND (human) |  | 50 |
| **Database: BIREME/LILACS** | | | |
| **Date: 24 October 2021** | | | |
| **Search** | **Strategy** | **Filters (if any)** | **Results** |
| 1 | (ab:(("climate change") OR ("climate disruption") OR ("climate hazard") OR ("climate extreme") OR ("climate catastrophe") OR ("climate shift") OR ("global warming") OR ("climate variation") OR ("climate variability") OR ("changing climate") OR ("weather change") OR ("meteorological change") OR ("temperature change") OR ("temperature extreme") OR ("temperature anomaly") OR ("rising temperature") OR ("temperature increase") OR ("temperature decrease") OR (heat-wave) OR (warm-spell) OR (cold-spell) OR ("extreme heat") OR ("extreme cold") OR ("high temperature") OR ("low temperature") OR (precipitation) OR ("humidity change") OR (mold) OR (moist) OR (evaporation) OR (muggy) OR (drought) OR ("water scarcity") OR ("water stress") OR (aridity) OR (dry-spell) OR ("water shortage") OR ("palmer drought severity index") OR (flood) OR (inundation) OR (deluge) OR (downpour) OR (rain) OR (alluvion) OR ("extreme weather event") OR ("extreme climate event") OR ("extreme meteorological event") OR ("extreme hydrometeorological event") OR (hurricane) OR (tornado) OR (typhoon) OR (storm) OR (windstorm) OR ("tropical cyclone") OR ("ocean acidification") OR ("sea acidification") OR ("ocean warming") OR ("ocean heating") OR ("sea heating") OR ("sea warming") OR ("el nino") OR ("el niño") OR ("sea surface temperature") OR (enso) OR ("southern oscillation") OR ("sea level rise") OR ("rising sea level") OR ("coastal erosion") OR ("algae bloom") OR ("brown tide") OR ("red tide") OR ("marine bloom") OR ("water bloom") OR ("black water") OR ("retreat of glacier") OR ("melting sea ice") OR ("glacial meltwater") OR ("glacier recession") OR ("reduced sea ice") OR ("ice sheet melting") OR ("glacier shrink") OR (biodiversity) OR ("biodiversity change") OR ("biodiversity loss") OR ("specie extintion") OR ("specie loss") OR ("specie disappearance") OR ("biological diversity loss") OR ("ecosystem change") OR ("vector suitability") OR ("vector adequacy") OR ("vector capacity") OR (wild-fire) OR (wildfire) OR (bush-fire) OR (bushfire) OR ("forest fire") OR ("wild land fire"))) AND (ab:((Exposure) OR (Exposition) OR (Exposed))) AND (ab:(("South America") OR (South-America) OR (argentina) OR (bolivia) OR (brazil) OR (chile) OR (colombia) OR (ecuador) OR (paraguay) OR (peru) OR (uruguay) OR (venezuela) OR (andes) OR (amazon) OR (altiplano) OR (suriname) OR (guyana))) AND (human) |  | 36 |

**Table S4.** Search strategies for: *What are the main vulnerabilities or susceptibility factors present in the South American human population groups that could increase the risk of climate change adverse impacts on health and wellbeing?*

| **Database: Web of Science** | | | |
| --- | --- | --- | --- |
| **Date: 10 October 2021** | | | |
| **Search** | **Strategy** | **Filters (if any)** | **Results** |
| 1 | TS=(“Climat* change$” OR “Climat* disrupt*” OR “Climat* hazard$” OR “Climat* extreme*” OR “Climat* catastroph*” OR “Climat* shift*” OR “Global warming” OR “Climat* variation$” OR “Climat* variab*” OR “Chang* climat*” OR “Weather change*” OR “Meteorol* chang*”) | None | 382,445 |
| 2 | TS=(“Temperature$ chang*” OR “Temperature* extreme$” OR “Temperature* anomal*” OR “Rising temperature*” OR “Temperature increase” OR “Temperature decrease” OR heat-wave OR Warm-spell OR Cold-spell OR “Extreme heat” OR “Extreme cold” OR “High temperature$” OR “Low temperature$”) | None | 841,421 |
| 3 | TS=(“Precipitation$” OR Moist OR Muggy OR Drought$ OR “Water scarcity” OR “Water stress” OR “Aridity” OR Dry-spell OR “Water Shortage$” OR “Palmer Drought Severity Index” OR Flood$ OR Inundation$ OR Deluge OR Downpour* OR Rain* OR Alluvion$) | None | 1,209,999 |
| 4 | TS=(“Extreme weather event$” OR “Extreme climat* event$” OR “Extreme hydrometeorological event$” OR “Extreme meteorological event$” OR Hurricane$ OR Tornado$ OR Typhoon$ OR Storm$ OR Windstorm$ OR “Tropical cyclone$”) | None | 139,900 |
| 5 | TS=(“Ocean acidification” OR “Sea acidification” OR “Ocean warming” OR “Ocean heat*” OR “El Ni$o” OR “Sea surface temperature” OR ENSO OR “ Southern Oscillation” OR “Sea level ris*” OR “Rising sea level*” OR “Coastal erosion” OR “Algae bloom*” OR “Brown tide” OR “Red tide” OR “Marine bloom” OR “Water bloom” OR “Black water” OR “Retreat of glacier*” OR “Melting sea ice” OR “Glacial meltwater*” OR “Glacier recession” OR “Reduced sea ice” OR “Ice sheet melt*” OR “Glacier shrink*” ) | None | 95,738 |
| 6 | TS=(biodiversity OR “Biodiversity change*” OR “Biodiversity loss*” OR “Specie* extinction” OR “Specie* loss” OR “Specie* disappearance” OR “Biological diversity loss*” OR “Ecosystem change*” OR “Vector* suitability” OR “Vector* adequacy” OR “Vector* capacity”) | None | 167,135 |
| 7 | TS=(Wild-fire$ OR Bush-fire$ OR “Forest fire$” OR “Wild-land fire*”) | None | 12,716 |
| 8 | TS=(health OR well-being OR quality-of-life OR welfare) | None | 3,156,161 |
| 9 | TS=(Death$ OR Mortal* OR Fatalit* OR Casualt* OR Disease$ OR Disorder$ OR Illness* OR Sickness* OR Indisposition$ OR Unhealth* OR Injur*) | None | 8,235,107 |
| 10 | TS=(Heat OR Heat-stroke$ OR Faintness OR “Warm skin” OR Dehydration OR Heat-stress OR “Heat exhaustion” OR “Heat hyperpyrexia” OR “Heat-related death$” OR “Respiratory disease$” OR “Vector-borne disease$” OR “Mosquito-borne Disease$” OR “Rodent-borne disease$” OR “Food-borne disease$” OR “Water-borne disease$” OR Poisoning) | None | 1,714,038 |
| 11 | TS=(“Food secur*” OR “Food scarcity” OR “Food shortage” OR “Food deprivation” OR “Food deficit” OR “Food shortfall” OR Malnutrit* OR Undernutri* OR Undernourish* OR Malnourish* OR Starv* OR Underfed OR Famine OR Hunger) | None | 184,790 |
| 12 | TS=(South-America* OR Argentin* OR Bolivia* OR Brazil* OR Chile* OR Colombia* OR Ecuador* OR Paraguay* OR Peru* OR Uruguay* OR Venezuela* OR Andes OR Amazon OR Altiplano OR Surinam* OR Guyan*) | None | 702,052 |
| 13 | TS=(Vulnerab* OR Susceptib*) | None | 1,026,648 |
| 14 | TS=(human) | None | - |
| 15 | (#1 OR #2 OR #3 OR #4 OR #5 OR #6 OR #7) AND (#8 OR #9 OR #10 OR #11) AND #13 AND #14 | None | 235 |
| **Database: PubMed** | | | |
| **Date: 24 October 2021** | | | |
| **Search** | **Strategy** | **Filters (if any)** | **Results** |
| 1 | “Climate change”[TIAB] OR “Climate changes”[TIAB] OR “Climatic change”[TIAB] OR “Climatic changes”[TIAB] OR “Climate disruption”[TIAB] OR “Climate disruptions”[TIAB] OR “Climatic disruption”[TIAB] OR “Climatic disruptions”[TIAB] OR “Climate hazard”[TIAB] OR “Climate hazards”[TIAB] OR “Climatic hazard”[TIAB] OR “Climatic hazards”[TIAB] OR “Climate extreme”[TIAB] OR “Climate extremes”[TIAB] OR “Climatic extreme”[TIAB] OR “Climatic extremes”[TIAB] OR “Climate catastrophe”[TIAB] OR “Climate catastrophes”[TIAB] OR “Climatic catastrophe”[TIAB] OR “Climatic catastrophes”[TIAB] OR “Climate shift”[TIAB] OR “Climate shifts”[TIAB] OR “Climatic shift”[TIAB] OR “Climatic shifts”[TIAB] OR “Global warming”[TIAB] OR “Climate variation”[TIAB] OR “Climate variations”[TIAB] OR “Climatic variation”[TIAB] OR “Climatic variations”[TIAB] OR “Climate variability”[TIAB] OR “Climate variabilities”[TIAB] OR “Climatic variability”[TIAB] OR “Climatic variabilities”[TIAB] OR “Changing Climate”[TIAB] OR “Weather change”[TIAB] OR “Weather changes”[TIAB] OR “Meteorological change”[TIAB] |  | 57,543 |
| 2 | “Temperature changes”[TIAB] OR “Temperature change”[TIAB] OR “Temperature changing”[TIAB] OR “Temperature extreme”[TIAB] OR “Temperature extremes”[TIAB] OR “Temperature anomaly”[TIAB] OR “Temperature anomalies”[TIAB] OR “Rising temperature”[TIAB] OR “Rising temperatures”[TIAB] OR “Temperature increase”[TIAB] OR “Temperature increasing”[TIAB] OR “Temperature decrease”[TIAB] OR “Temperature decreasing”[TIAB] OR heat-wave[TIAB] OR heatwave[TIAB] OR “heat wave”[TIAB] OR warmspell[TIAB] OR warm-spell[TIAB] OR “Warm spell”[TIAB] OR “Warm spells”[TIAB] OR coldspell[TIAB] OR cold-spell[TIAB] OR “Cold spell”[TIAB] OR “Cold spells”[TIAB] OR “Extreme heat”[TIAB] OR “Extreme cold”[TIAB] OR “High temperature”[TIAB] OR “High temperatures”[TIAB] OR “Higher temperatures”[TIAB] OR “Higher temperature”[TIAB] OR “Low temperature”[TIAB] OR “Low temperatures”[TIAB] OR “Lower temperatures”[TIAB] OR “Lower temperature”[TIAB] |  | 143,297 |
| 3 | Precipitati*[TIAB] OR Moist*[TIAB] OR Evaporation[TIAB] OR Muggy[TIAB] OR Drought*[TIAB] OR “Water scarcity”[TIAB] OR “Water stress”[TIAB] OR “Aridity”[TIAB] OR dry-spell[TIAB] OR “Dry spell”[TIAB] OR “Water Shortage”[TIAB] OR “Palmer Drought Severity Index”[TIAB] OR Flood*[TIAB] OR Inundation*[TIAB] OR Deluge[TIAB] OR Downpour*[TIAB] OR Rain*[TIAB] OR Alluvion*[TIAB] |  | 247,308 |
| 4 | “Extreme weather event”[TIAB] OR “Extreme weather events”[TIAB] OR “Extreme climate event”[TIAB] OR “Extreme climatic event”[TIAB] OR “Extreme climate events”[TIAB] OR “Extreme climate event”[TIAB] OR “Extreme hydrometeorological event”[TIAB] OR “Extreme meteorological events”[TIAB] OR Hurricane*[TIAB] OR Tornado*[TIAB] OR Typhoon*[TIAB] OR Storm*[TIAB] OR Windstorm*[TIAB] OR “Tropical cyclone”[TIAB] OR “Tropical cyclones”[TIAB] |  | 25,122 |
| 5 | “Ocean acidification”[TIAB] OR “Ocean warming”[TIAB] OR “Ocean heat”[TIAB] OR “Ocean heating”[TIAB] OR “El Nino”[TIAB] OR “El Niño”[TIAB] OR “Sea surface temperature”[TIAB] OR ENSO[TIAB] OR “Southern Oscillation”[TIAB] OR “Sea level rise”[TIAB] OR “Sea level rising”[TIAB] OR “Rising sea level”[TIAB] OR “Rising sea levels”[TIAB] OR “Coastal erosion”[TIAB] OR “Algae bloom”[TIAB] OR “Brown tide”[TIAB] OR “Red tide”[TIAB] OR “Marine bloom”[TIAB] OR “Water bloom”[TIAB] OR “Black water”[TIAB] OR “Retreat of glacier”[TIAB] OR “Retreat of glaciers”[TIAB] OR “Melting sea ice”[TIAB] OR “Glacial meltwater”[TIAB] OR “Glacier recession”[TIAB] OR “Reduced sea ice”[TIAB] OR “Ice sheet melt”[TIAB] OR “Ice sheet melting”[TIAB] OR “Glacier shrinking”[TIAB] OR “Glacier shrinkage”[TIAB] |  | 9,203 |
| 6 | biodiversity[TIAB] OR “Biodiversity change”[TIAB] OR “Biodiversity changes”[TIAB] OR “Biodiversity loss”[TIAB] OR “Specie extinction”[TIAB] OR “Species extinction”[TIAB] OR “Specie extinctions”[TIAB] OR “Species extinctions”[TIAB] OR “Specie loss”[TIAB] OR “Species loss”[TIAB] OR “Specie disappearance”[TIAB] OR “Species disappearance”[TIAB] OR “Biological diversity loss” [TIAB] OR “Ecosystem change”[TIAB] OR “Ecosystem changes”[TIAB] OR “Vector suitability”[TIAB] OR “Vector adequacy”[TIAB] OR “Vector capacity”[TIAB] OR “vectorial suitability”[TIAB] OR “vectorial adequacy”[TIAB] OR “vectorial capacity”[TIAB] |  | 35,054 |
| 7 | “Wild fire”[TIAB] OR “Wild fires”[TIAB] OR wild-fire*[TIAB] OR Wildfire*[TIAB] OR “Bush fire”[TIAB] OR “Bush fires”[TIAB] OR bushfire*[TIAB] OR bush-fire*[TIAB] OR “Forest fire”[TIAB] OR “Forest fires”[TIAB] OR “Wild-land fire”[TIAB] OR “Wild-land fires”[TIAB] |  | 3,956 |
| 8 | Vulnerab*[TIAB] OR Susceptib*[TIAB] |  | 601,284 |
| 9 | South-America*[TIAB] OR South America*[TIAB] OR Argentin*[TIAB] OR Bolivia*[TIAB] OR Brazil*[TIAB] OR Chile*[TIAB] OR Colombia*[TIAB] OR Ecuador*[TIAB] OR Paraguay*[TIAB] OR Peru*[TIAB] OR Uruguay*[TIAB] OR Venezuela*[TIAB] OR Ande*[TIAB] OR Amazon[TIAB] OR Altiplano[TIAB] OR Surinam*[TIAB] OR Guyan*[TIAB] |  | 251,703 |
| 10 | #2 OR #3 OR #4 OR #5 OR #6 OR #7 |  | 443,940 |
| 11 | #1 AND #8 AND #9 AND #10 | Humans | 131 |
| **Database: Pubmed MeSH** | | | |
| **Date: 24 October 2021** | | | |
| **Search** | **Strategy** | **Filters (if any)** | **Results** |
| 1 | ("Global Warming"[Mesh]) OR "Climate Change"[Mesh] |  | 23,906 |
| 2 | ((((("Extreme Heat"[Mesh]) OR "Cold Temperature"[Mesh]) OR "Hot Temperature"[Mesh]) OR "Extreme Hot Weather"[Mesh]) OR "Extreme Cold Weather"[Mesh]) OR "Extreme Weather"[Mesh] |  | 190,930 |
| 3 | (((("Rain"[Mesh]) OR "Humidity"[Mesh]) OR "Droughts"[Mesh]) OR "Floods"[Mesh]) OR "Water Insecurity"[Mesh] |  | 40,864 |
| 4 | (("Extreme Weather"[Mesh]) OR "Cyclonic Storms"[Mesh]) OR "Tornadoes"[Mesh] |  | 2,982 |
| 5 | (("El Nino-Southern Oscillation"[Mesh]) OR "Sea Level Rise"[Mesh]) OR "Harmful Algal Bloom"[Mesh] |  | 1,880 |
| 6 | ("Biodiversity"[Mesh]) OR "Extinction, Biological"[Mesh] |  | 104,059 |
| 7 | "Wildfires"[Mesh] |  | 702 |
| 8 | (("Risk"[Mesh]) OR "Vulnerable Populations"[Mesh]) OR "Disease Susceptibility"[Mesh] |  | 193,797 |
| 9 | (((((((((((("South America"[Mesh]) OR "Argentina"[Mesh]) OR "Bolivia"[Mesh]) OR "Brazil"[Mesh]) OR "Chile"[Mesh]) OR "Colombia"[Mesh]) OR "Ecuador"[Mesh]) OR "Paraguay"[Mesh]) OR "Peru"[Mesh]) OR "Suriname"[Mesh]) OR "Uruguay"[Mesh]) OR "Venezuela"[Mesh]) OR "Guyana"[Mesh] |  | 180,741 |
| 10 | #2 OR #3 OR #4 OR #5 OR #6 OR #7 |  | 335,061 |
| 11 | #1 AND #8 AND #9 AND #10 | Humans | 3 |
| **Database: ProQuest** | | | |
| **Date: 24 October 2021** | | | |
| **Search** | **Strategy** | **Filters (if any)** | **Results** |
|  | ab("Climat* change$" OR "Climat* disrupt*" OR "Climat* hazard$" OR "Climat* extreme*" OR "Climat* catastroph*" OR "Climat* shift*" OR "Global warming" OR "Climat* variation$" OR "Climat* variab*" OR "Chang* climat*" OR ("weather change" OR "weather changed" OR "weather changes") OR "Meteorol* chang*" OR "Temperature$ chang*" OR "Temperature* extreme$" OR "Temperature* anomal*" OR ("rising temperature" OR "rising temperatures") OR "Temperature increase" OR "Temperature decrease" OR heat-wave OR Warm-spell OR Cold-spell OR "Extreme heat" OR "Extreme cold" OR "High temperature$" OR "Low temperature$" OR "Precipitation$" OR "Humid* chang*" OR Mold OR Moist OR Evaporation OR Muggy OR Drought OR "Water scarcity" OR "Water stress" OR "Aridity" OR Dry-spell OR "Water Shortage$" OR "Palmer Drought Severity Index" OR Flood OR Inundation OR Deluge OR Downpour* OR Rain* OR Alluvion OR "Extreme weather event$" OR "Extreme climat* event$" OR "Extreme hydrometeorological event$" OR "Extreme meteorological event$" OR Hurricane OR Tornado OR Typhoon OR Storm OR Windstorm OR "Tropical cyclone$" OR "Ocean acidification" OR "Sea acidification" OR "Ocean warming" OR ("ocean heat") OR "Sea heat*" OR "Sea warming" OR "El Ni$o" OR "Sea surface temperature" OR ENSO OR " Southern Oscillation" OR "Sea level ris*" OR "Rising sea level*" OR "Coastal erosion" OR ("algae bloom" OR "algae blooms") OR "Brown tide" OR "Red tide" OR "Marine bloom" OR "Water bloom" OR "Black water" OR "Retreat of glacier*" OR "Melting sea ice" OR ("glacial meltwater" OR "glacial meltwaters") OR "Glacier recession" OR "Reduced sea ice" OR "Ice sheet melt*" OR "Glacier shrink*" OR biodiversity OR "Biodiversity change*" OR ("biodiversity loss") OR "Specie* extinction" OR "Specie* loss" OR "Specie* disappearance" OR "Biological diversity loss*" OR ("ecosystem change" OR "ecosystem changes") OR "Vector* suitability" OR "Vector* adequacy" OR "Vector* capacity" OR Wild-fire OR Bush-fire OR "Forest fire$" OR "Wild-land fire*") AND ab(Vulnerab* OR Susceptib*) AND ab(South-America* OR Argentin* OR Bolivia* OR Brazil* OR Chile* OR Colombia* OR Ecuador* OR Paraguay* OR Peru* OR Uruguay* OR Venezuela* OR Andes OR Amazon OR Altiplano OR Surinam* OR Guyan*) AND ab(human) |  | 231 |
| **Database: Scopus** | | | |
| **Date: 24 October 2021** | | | |
| **Search** | **Strategy** | **Filters (if any)** | **Results** |
|  | ( TITLE-ABS-KEY ( {Climate change} OR {Climate disruption} OR {Climate hazard} OR {Climate extreme} OR {Climate catastrophe} OR {Climate shift} OR {Global warming} OR {Climate variation} OR {Climate variability} OR {Changing climate} OR {Weather change} OR {Meteorological change} OR {Temperature change} OR {Temperature extreme} OR {Temperature anomaly} OR {Rising temperature} OR {Temperature increase} OR {Temperature decrease} OR heat-wave OR warm-spell OR cold-spell OR {Extreme heat} OR {Extreme cold} OR {High temperature} OR {Low temperature} OR precipitation* OR {Humidity change} OR mold OR moist OR evaporation OR muggy OR drought* OR {Water scarcity} OR {Water stress} OR aridity OR dry-spell OR {Water Shortage} OR {Palmer Drought Severity Index} OR flood* OR inundation* OR deluge OR downpour* OR rain* OR alluvion* OR {Extreme weather event} OR {Extreme climate event} OR {Extreme hydrometeorological event} OR {Extreme meteorological event} OR hurricane* OR tornado* OR typhoon* OR storm* OR windstorm* OR {Tropical cyclone} OR {ocean acidification} OR {sea acidification} OR {ocean warming} OR {ocean heating} OR {sea heating} OR {sea warming} OR {el nino} OR {el niño} OR {sea surface temperature} OR enso OR {southern oscillation} OR {sea level rise} OR {rising sea level} OR {coastal erosion} OR {algae bloom} OR {brown tide} OR {red tide} OR {marine bloom} OR {water bloom} OR {black water} OR {retreat of glacier} OR {melting sea ice} OR {glacial meltwater} OR {glacier recession} OR {reduced sea ice} OR {ice sheet melting} OR {glacier shrink} OR biodiversity OR {Biodiversity change} OR {Biodiversity loss} OR {Specie extinction} OR {Specie loss} OR {Specie disappearance} OR {Biological diversity loss} OR {Ecosystem change} OR {Vector suitability} OR {Vector adequacy} OR {Vector capacity} OR wild-fire OR wildfire OR bush-fire OR bushfire OR {Forest fire} OR {Wild-land fire} ) AND TITLE-ABS-KEY ( "South-America*" OR argentin* OR bolivia* OR brazil* OR chile* OR colombia* OR ecuador* OR paraguay* OR peru* OR uruguay* OR venezuela* OR andes OR amazon OR altiplano OR surinam* OR guyan* ) AND TITLE-ABS-KEY ( vulnerab* OR susceptib* ) ) AND ( LIMIT-TO ( SUBJAREA , "ENVI" ) OR LIMIT-TO ( SUBJAREA , "MEDI" ) OR LIMIT-TO ( SUBJAREA , "NURS" ) OR LIMIT-TO ( SUBJAREA , "PSYC" ) OR LIMIT-TO ( SUBJAREA , "HEAL" ) ) AND ( LIMIT-TO ( EXACTKEYWORD , "Human" ) ) |  | 226 |
| **Database: SciELO** | | | |
| **Date: 24 October 2021** | | | |
| **Search** | **Strategy** | **Filters (if any)** | **Results** |
| 1 | ((("climate change" OR "climate disruption" OR "climate hazard" OR "climate extreme" OR "climate catastrophe" OR "climate shift" OR "global warming" OR "climate variation" OR "climate variability" OR "changing climate" OR "weather change" OR "meteorological change") OR ("temperature change" OR "temperature extreme" OR "temperature anomaly" OR "rising temperature" OR "temperature increase" OR "temperature decrease" OR heat-wave OR warm-spell OR cold-spell OR "extreme heat" OR "extreme cold" OR "high temperature" OR "low temperature") OR (precipitation OR "humidity change" OR mold OR moist OR evaporation OR muggy OR drought OR "water scarcity" OR "water stress" OR aridity OR dry-spell OR "water shortage" OR "palmer drought severity index" OR flood OR inundation OR deluge OR downpour OR rain OR alluvion) OR ("extreme weather event" OR "extreme climate event" OR "extreme meteorological event" OR "extreme hydrometeorological event" OR hurricane OR tornado OR typhoon OR storm OR windstorm OR "tropical cyclone") OR ("ocean acidification" OR "sea acidification" OR "ocean warming" OR "ocean heating" OR "sea heating" OR "sea warming" OR "el nino" OR "el niño" OR "sea surface temperature" OR enso OR "southern oscillation" OR "sea level rise" OR "rising sea level" OR "coastal erosion" OR "algae bloom" OR "brown tide" OR "red tide" OR "marine bloom" OR "water bloom" OR "black water" OR "retreat of glacier" OR "melting sea ice" OR "glacial meltwater" OR "glacier recession" OR "reduced sea ice" OR "ice sheet melting" OR "glacier shrink") OR (biodiversity OR "biodiversity change" OR "biodiversity loss" OR "specie extinction" OR "specie loss" OR "specie disappearance" OR "biological diversity loss" OR "ecosystem change" OR "vector suitability" OR "vector adequacy" OR "vector capacity") OR (wild-fire OR wildfire OR bush-fire OR bushfire OR "forest fire" OR "wild land fire")) AND (vulnerab* OR susceptib*) AND ("South America" OR South-America* OR argentin* OR bolivia* OR brazil* OR chile* OR colombia* OR ecuador* OR paraguay* OR peru* OR uruguay* OR venezuela* OR andes OR amazon OR altiplano OR surinam* OR guyan*)) AND (human) |  | 64 |
| **Database: BIREME/LILACS** | | | |
| **Date: 24 October 2021** | | | |
| **Search** | **Strategy** | **Filters (if any)** | **Results** |
| 1 | (ab:(("climate change") OR ("climate disruption") OR ("climate hazard") OR ("climate extreme") OR ("climate catastrophe") OR ("climate shift") OR ("global warming") OR ("climate variation") OR ("climate variability") OR ("changing climate") OR ("weather change") OR ("meteorological change") OR ("temperature change") OR ("temperature extreme") OR ("temperature anomaly") OR ("rising temperature") OR ("temperature increase") OR ("temperature decrease") OR (heat-wave) OR (warm-spell) OR (cold-spell) OR ("extreme heat") OR ("extreme cold") OR ("high temperature") OR ("low temperature") OR (precipitation) OR ("humidity change") OR (mold) OR (moist) OR (evaporation) OR (muggy) OR (drought) OR ("water scarcity") OR ("water stress") OR (aridity) OR (dry-spell) OR ("water shortage") OR ("palmer drought severity index") OR (flood) OR (inundation) OR (deluge) OR (downpour) OR (rain) OR (alluvion) OR ("extreme weather event") OR ("extreme climate event") OR ("extreme meteorological event") OR ("extreme hydrometeorological event") OR (hurricane) OR (tornado) OR (typhoon) OR (storm) OR (windstorm) OR ("tropical cyclone") OR ("ocean acidification") OR ("sea acidification") OR ("ocean warming") OR ("ocean heating") OR ("sea heating") OR ("sea warming") OR ("el nino") OR ("el niño") OR ("sea surface temperature") OR (enso) OR ("southern oscillation") OR ("sea level rise") OR ("rising sea level") OR ("coastal erosion") OR ("algae bloom") OR ("brown tide") OR ("red tide") OR ("marine bloom") OR ("water bloom") OR ("black water") OR ("retreat of glacier") OR ("melting sea ice") OR ("glacial meltwater") OR ("glacier recession") OR ("reduced sea ice") OR ("ice sheet melting") OR ("glacier shrink") OR (biodiversity) OR ("biodiversity change") OR ("biodiversity loss") OR ("specie extinction") OR ("specie loss") OR ("specie disappearance") OR ("biological diversity loss") OR ("ecosystem change") OR ("vector suitability") OR ("vector adequacy") OR ("vector capacity") OR (wild-fire) OR (wildfire) OR (bush-fire) OR (bushfire) OR ("forest fire") OR ("wild land fire"))) AND (ab:((vulnerability) OR (susceptibility))) AND (ab:(("South America") OR (South-America) OR (argentina) OR (bolivia) OR (brazil) OR (chile) OR (colombia) OR (ecuador) OR (paraguay) OR (peru) OR (uruguay) OR (venezuela) OR (andes) OR (amazon) OR (altiplano) OR (suriname) OR (guyana))) |  | 41 |
