# Supplementary material for: Identifying gaps on health impacts, exposures, and vulnerabilities to climate change on human health and wellbeing in South America: a scoping review
Source: Lancet Reg Health Am. 2023 Aug 24;26:100580. doi: 10.1016/j.lana.2023.100580 (PMC10593580; doi:10.1016/j.lana.2023.100580)
Supplement: Translated summary_ESP - disclaimer ok [file mmc2.docx]

**Editorial disclaimer:** “*This translation in Spanish was submitted by the authors and we reproduce it as supplied. It has not been peer reviewed. Our editorial processes have only been applied to the original abstract in English, which should serve as reference for this manuscript.*

**Resumen**

Existe una brecha importante en la información regional sobre el cambio climático y la salud, lo que limita el desarrollo de políticas climáticas basadas en la ciencia en los países de América del Sur. Este estudio tiene como objetivo identificar las principales brechas en la literatura científica existente sobre los impactos, la exposición y las vulnerabilidades del cambio climático en la salud de la población. Se realizó una revisión de alcance guiada por cuatro sub-preguntas centradas en los impactos del cambio climático en la salud física y mental, los factores de exposición y vulnerabilidad de la población ante los peligros climáticos. Los principales hallazgos mostraron que los impactos físicos incluyeron principalmente enfermedades infecciosas, mientras que los impactos en la salud mental incluyeron trauma, depresión y ansiedad. La evidencia sobre la exposición de la población a los peligros climáticos es limitada, y se identificaron los determinantes sociales de la salud y los factores individuales como factores de vulnerabilidad. En general, la evidencia sobre la intersección entre el cambio climático y la salud es limitada en América del Sur y se ha generado de manera aislada, con una investigación transdisciplinaria limitada. Se debe generar más información formal y sistemática para informar las políticas públicas.
