# Supplementary material for: Identifying gaps on health impacts, exposures, and vulnerabilities to climate change on human health and wellbeing in South America: a scoping review
Source: Lancet Reg Health Am. 2023 Aug 24;26:100580. doi: 10.1016/j.lana.2023.100580 (PMC10593580; doi:10.1016/j.lana.2023.100580)
Supplement: Translated summary_POR - disclaimer ok [file mmc3.docx]

**Editorial disclaimer:** “*This translation in Portuguese was submitted by the authors and we reproduce it as supplied. It has not been peer reviewed. Our editorial processes have only been applied to the original abstract in English, which should serve as reference for this manuscript.*

**Resumo**

Existe uma lacuna importante na informação regional sobre mudanças climáticas e saúde, o que limita o desenvolvimento de políticas climáticas baseadas em ciência nos países sul-americanos. Este estudo tem como objetivo identificar as principais lacunas na literatura científica existente sobre os impactos, exposição e vulnerabilidades das mudanças climáticas na saúde da população. Foi realizada uma revisão de escopo orientada por quatro subperguntas focadas nos impactos das mudanças climáticas na saúde física e mental, fatores de exposição e vulnerabilidade da população aos riscos climáticos. Os principais resultados mostraram que os impactos físicos incluíam principalmente doenças infecciosas, enquanto os impactos na saúde mental incluíam trauma, depressão e ansiedade. A evidência sobre a exposição da população aos riscos climáticos é limitada, e os determinantes sociais da saúde e os fatores individuais foram identificados como fatores de vulnerabilidade. No geral, a evidência sobre a interseção entre mudanças climáticas e saúde é limitada na América do Sul e tem sido gerada de forma isolada, com pesquisas transdisciplinares limitadas. Deve-se gerar mais informações formais e sistemáticas para subsidiar as políticas públicas.
